# Plasticity of photoreceptor-generating retinal progenitors revealed by prolonged retinoic acid exposure

**DOI:** 10.1186/1471-213X-11-51

**Published:** 2011-08-30

**Authors:** Craig B Stevens, David A Cameron, Deborah L Stenkamp

**Affiliations:** 1 Department of Biological Sciences, University of Idaho, Moscow ID 83844, USA; 2 Department Neuroscience and Physiology, SUNY Upstate Medical University, Syracuse, NY 13210, USA

## Abstract

**Background:**

Retinoic acid (RA) is important for vertebrate eye morphogenesis and is a regulator of photoreceptor development in the retina. In the zebrafish, RA treatment of postmitotic photoreceptor precursors has been shown to promote the differentiation of rods and red-sensitive cones while inhibiting the differentiation of blue- and UV-sensitive cones. The roles played by RA and its receptors in modifying photoreceptor fate remain to be determined.

**Results:**

Treatment of zebrafish embryos with RA, beginning at the time of retinal progenitor cell proliferation and prior to photoreceptor terminal mitosis, resulted in a significant alteration of rod and cone mosaic patterns, suggesting an increase in the production of rods at the expense of red cones. Quantitative pattern analyses documented increased density of rod photoreceptors and reduced local spacing between rod cells, suggesting rods were appearing in locations normally occupied by cone photoreceptors. Cone densities were correspondingly reduced and cone photoreceptor mosaics displayed expanded and less regular spacing. These results were consistent with replacement of approximately 25% of positions normally occupied by red-sensitive cones, with additional rods. Analysis of embryos from a RA-signaling reporter line determined that multiple retinal cell types, including mitotic cells and differentiating rods and cones, are capable of directly responding to RA. The RA receptors RXRγ and RARαb are expressed in patterns consistent with mediating the effects of RA on photoreceptors. Selective knockdown of RARαb expression resulted in a reduction in endogenous RA signaling in the retina. Knockdown of RARαb also caused a reduced production of rods that was not restored by simultaneous treatments with RA.

**Conclusions:**

These data suggest that developing retinal cells have a dynamic sensitivity to RA during retinal neurogenesis. In zebrafish RA may influence the rod vs. cone cell fate decision. The RARαb receptor mediates the effects of endogenous, as well as exogenous RA, on rod development.

## Background

The vertebrate retina forms from a neuroepithelium that develops into a complex, layered structure of neurons consisting of the ganglion cell layer (GCL); the inner nuclear layer (INL), composed of the amacrine, horizontal, and bipolar cells; and the outer nuclear layer (ONL), composed of the light-sensing photoreceptors. The retinal photoreceptor layer is apposed by the non-neuronal layer of retinal pigmented epithelial (RPE) cells. Retinal neurogenesis follows a common pattern in most species; in zebrafish the ganglion cells are the first to become postmitotic, followed by the cells of the INL [1]. The last neurons to be generated and then differentiate are the photoreceptors [1]. The photoreceptor mosaic of teleost fish, such as zebrafish, forms a spatially regular pattern of rods and cones [2-5].

The signaling pathways that regulate the production of rod and cone photoreceptors into their regular spatial patterns are not well understood. In the larval and adult teleost, rod and cone neurogenesis are spatially distinct, with new cones generated from stem cells residing in a circumferential germinal zone (CGZ), and new rods arising from a proliferative lineage residing within the INL [6-9]. There is evidence that Müller glia constitute the apex of the rod lineage, remaining proliferative and generating progeny that migrate to the ONL, undergo terminal mitoses, and differentiate as rods [10-12]. Despite apparently distinct lineage histories of rods and cones, the two types of progenitor cells are, at the molecular level, virtually indistinguishable, and express several photoreceptor-specific transcription factors including *crx, rx1, neuroD, nrl*, and *nr2e3 *[11]. Furthermore, in zebrafish that are mutant for the *tbx2b *gene, encoding a transcription factor expressed in early retinal progenitors, the UV cones are conspicuously missing from the larval cone mosaic, their positions instead occupied by supernumerary rod photoreceptors [13], suggesting an alteration in cell fate choice by retinal progenitors. Together these findings suggest some overlap of, or plasticity within, the progenitor cell populations otherwise fated to generate rods or cones.

The development of retinal cells, including photoreceptors, is known to be controlled by a variety of secreted signaling factors, including retinoic acid (RA). RA and its receptors are essential for morphogenesis of the vertebrate eye. A deficiency of RA or its precursor Vitamin A leads to ocular defects such as coloboma and retinal dysplasia [14-18]. RA signaling occurs via structural dimers formed by one member each of the Retinoic Acid Receptor (RAR) and Retinoid × Receptor (RXR) subtypes [19-22]. In the chick and mouse, specific RARs and RXRs are expressed in cells of the INL, ONL, and the RPE, in overlapping and non-overlapping patterns [23,24]. Mouse embryos deficient in combinations of RAR/RXR genes exhibit defects in eye morphogenesis, including thinning of the retinal layers, targeted defects in the ventral retina, and absence of an ONL [25,26]. RA synthesis in the retina occurs in specific ventral and dorsal domains, defined by the expression of retinaldehyde dehydrogenases (RALDHs) [27-31] with boundaries formed by expression of cyp26 enzymes involved in RA degradation [32,33]. Studies using reporter lines to monitor RA signaling in the eye have also shown RA signaling to be dynamic, occurring initially in the ventral retina and later spreading to other parts of the retina [31,34,35].

The results from previous *in vitro *and *in vivo *studies suggested that RA can control the formation of photoreceptors. RA promotes the formation and survival of rod photoreceptors from cultured retinal progenitors, within the developing rat retina [36-38], and from mammalian embryonic stem cells [39]. RA signaling also controls the expression of photoreceptor-specific genes, such as the transcription factor NRL and opsin genes, the latter involving differential effects upon specific rod and cone opsins [31,36,38,40-43]. In addition, RA can restore the expression of photoreceptor-specific markers in embryos treated with ethanol [44]. Collectively these data indicate a significant contribution of RA signaling to the differentiation of rod and cone photoreceptors.

In this study, we tested the hypothesis that sustained high RA signaling during retinal neurogenesis modifies photoreceptor cell fate decisions. Although our previous study suggested that RA selectively regulates expression of opsin genes, the experimental design may have precluded a capacity to observe effects on fate because RA levels were selectively manipulated at the time of, or shortly after, photoreceptor terminal mitoses [31]. In the present study RA signaling was altered at an earlier stage of retinal development, at a time when the retinal progenitor sources of photoreceptors remain proliferative [1]. We report that prolonged RA treatment beginning prior to photoreceptor neurogenesis causes photoreceptor pattern abnormalities consistent with replacement of some cones in the mosaic with rods. An RA signaling reporter line was used to demonstrate that multiple cell types, including progenitor cells, and rod and cone photoreceptors, are directly responsive to changes in RA signaling. Knockdown of a specific receptor, RARαb, resulted in a reduction in the level of endogenous RA signaling and a decrease in the number of rod photoreceptors, suggesting this receptor plays a role in mediating the photoreceptor development function of endogenous RA signaling. In addition, the combination of RARαb knockdown and RA treatment did not restore rod photoreceptor differentiation, suggesting this receptor also plays a role in mediating the effects of exogenous RA.

## Results

### Sustained RA treatment beginning at the time of early retinal neurogenesis changes the ratio of rods to cones

**Figure 1:**
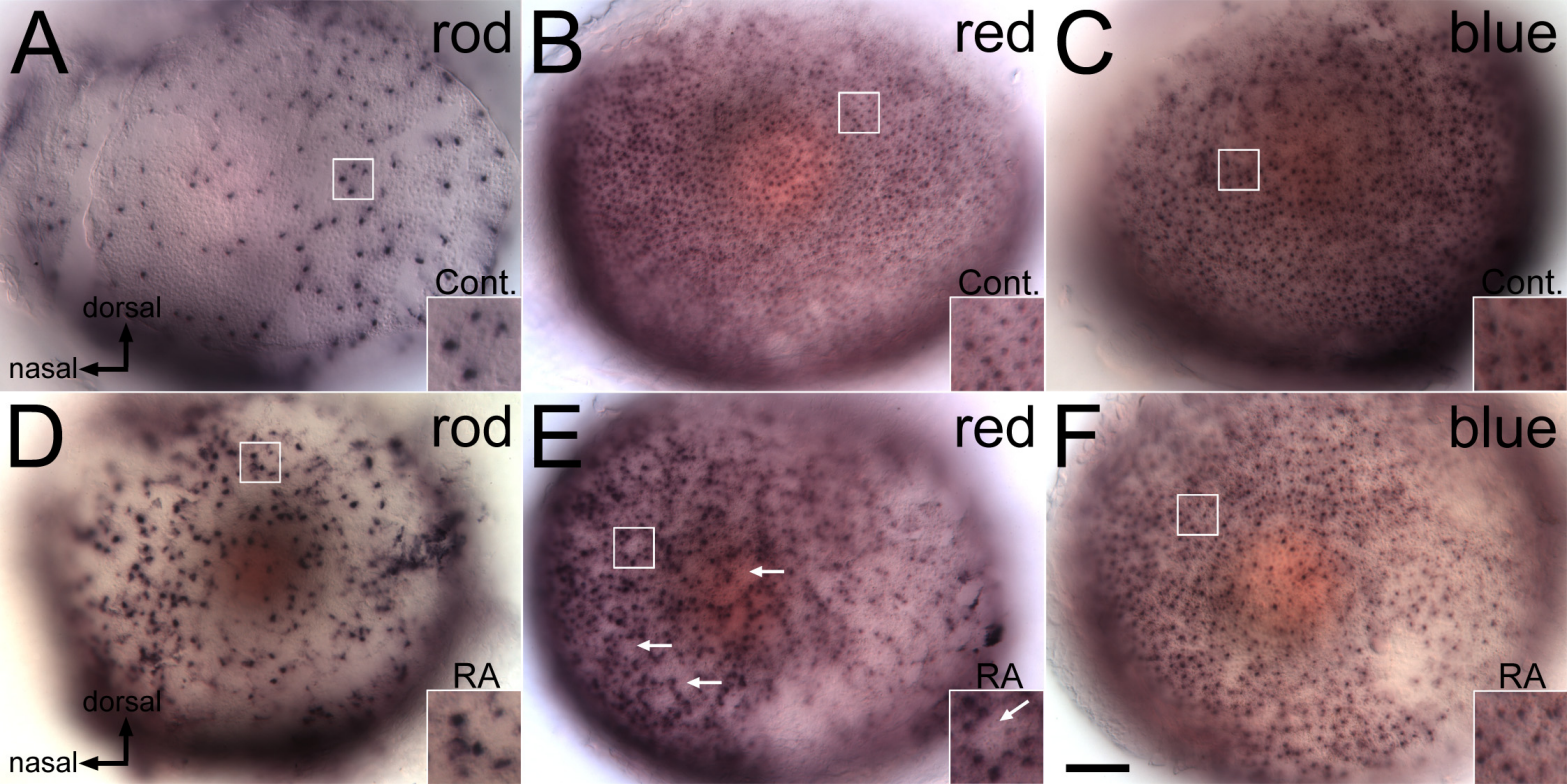
**Opsin expression is altered by retinoic acid treatment beginning at the time of retinal neurogenesis**. Embryos were treated with DMSO (Cont.; A, B, C), or 0.3 μM RA (D, E, F) at 36 hpf, and at 60 hpf were hybridized as whole mounts with probes corresponding to rod opsin (A, D), red cone opsin (B, E), or blue cone opsin (C, F); views are of whole embryonic eyes; dorsal is up and nasal to the left. In the control (DMSO-treated) embryos, rod photoreceptors are found predominantly in the ventral and dorsal regions (A), while red and blue cones (B, C) are evenly spread across the retina. In RA-treated embryos there is an apparent increase in rods, particularly in central regions of the retina (B), and a decrease in the appearance of red cones, leading to empty patches in the red cone mosaic (E, arrows). Boxed regions in each panel appear at higher magnification in the insets. Bar = 50 μm.

RA manipulations were performed beginning at 36 hpf, a time when much of the embryonic retina remains proliferative and no photoreceptors are definitively postmitotic [1]. Photoreceptor fates were determined by analyzing expression of rod and cone opsin mRNAs. In control embryos, rod photoreceptors first appear in the dorsal and ventral parts of the retina, with fewer rods found in the central retina (Figure 1A). Treatment with RA beginning at 36 hpf caused a significant increase in the density of rod opsin^+ ^photoreceptors (Figure 1D,Table 1). In contrast, RA treatment at 36 hpf resulted in a significant decrease in the density of cone photoreceptors expressing red and blue opsins (Figure 1B, C, E, F; Table 1), or UV opsins (Table 1 and data not shown). The red cone mosaic in particular displayed a disrupted pattern, with gaps suggesting that some red cones were missing from the normal mosaic (Figure 1B, E, arrows). Blue and UV cone mosaics were also affected, though to a lesser extent than that of red cones (Figure 1C, F, and data not shown). It should be noted that at the time of analysis (60 hpf), photoreceptor differentiation is not entirely complete in the ventrotemporal retina, resulting in a reduced level of opsin staining in these regions in some specimens. However, the unusual red cone mosaic pattern illustrated in Figure 1E was not limited to the nasal and dorsal parts of the retina. These results build upon those of a previous study [44] suggesting altered distribution of rods and red and green cones in response to RA treatment during retinal neurogenesis.

**Table 1:** Densities of rod and cone photoreceptors in control and RA-treated embryos, and in theoretical datasets

	Cell densities (cells/3600 μm^2^)			
	60 hpf		75 hpf	
**Cell Type**	**Control**	**RA**	**Control**	**RA**

Rods	9.56 ± 2.55	19.2 ± 6.75**	16.2 ± 2.68	N.D.^a^
Cones:				
Red	70.5 ± 12.69	53.33 ± 13.42*	59.50 ± 8.23	28.40 +8.53***
Blue	45.44 ± 5.66	37.1 ± 8.30*	52.56 ± 18.56	38.33 ± 10.71*
UV	47.33 ± 6.96	40.11 ± 5.80*	N.D.	N.D
	**Theoretical red cone loss (n = 5)**^**b**^			
	**25% loss**	**50% loss**	**25% loss**	**50% loss**
	
	48.4 ± 10.81**	32.40 ± 7.5***	43.20 ± 7.53**	29.40 ± 5.81***

*p < 0.05; **p < 0.01; ***p < 0.001 (Student's t-test).

N.D. = Not done.

^a ^Rod density was so high as to preclude accurate counting.

^b ^As compared to red cone density at 75 hpf.

**Figure 2:**
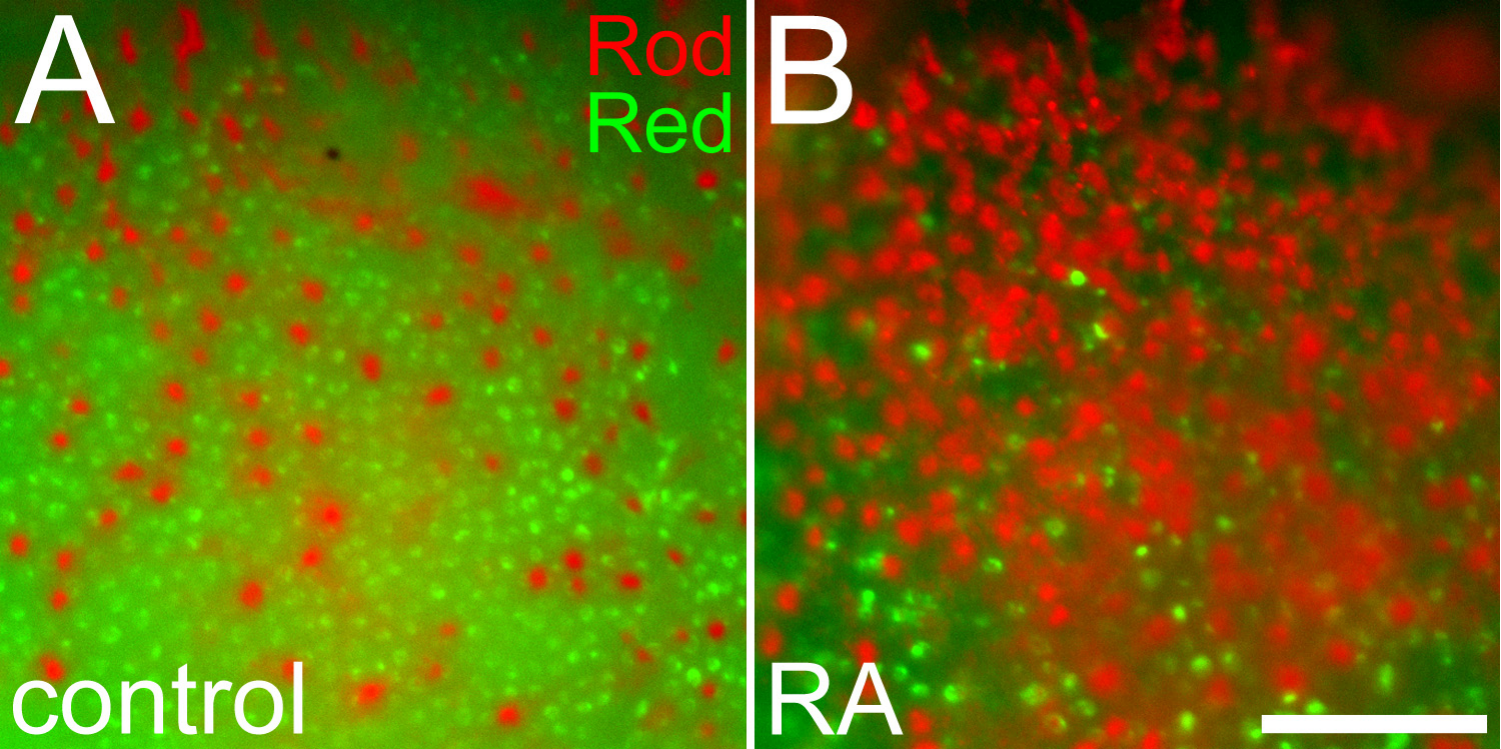
**Altered positioning of rods in relation to red cones in response to retinoic acid treatment**. Embryos treated with DMSO (A) or 0.3 μM RA (B) at 36 hpf and fixed at 60 hpf were doubly hybridized as whole mounts with probes corresponding to rod opsin (red color) and red cone opsin (green color). Following RA treatment, rods are more abundant, cones are more sparsely distributed, and rods display spacing characteristics more typical of cones. Bar = 50 μm.

We next performed fluorescent double in situ hybridization to visualize the number and position of rods and red cones simultaneously. This approach also detected the disrupted rod and red cone photoreceptor patterns in eyes of RA-treated embryos (Figure 2). Using the doubly-hybridized material, we measured a greater density of rods and a significantly lower density of red cones in embryos treated with RA during retinal neurogenesis (Table 2). However, the difference in rod density was not statistically significant. This may be related to differential sensitivities of the dual vs. single in situ hybridization approach to detect rods or cones.

**Table 2:** Densities of rods and red cones in retinas probed for both rod and red cone opsins

	Cell densities (cells/3600 μm^2^) 60 hpf		Theoretical red to rod **photoreceptor change (n = 6)**^**a**^	
**Cell Type**	**Control**	**RA**	**25%**	**50%**

Rods	13.3 ± 4.0	16.9 ± 4.3	25.2 ± 4.9	37.2 ± 5.4
Cones:				
Red	47.7 ± 8.2	35.8 ± 6.3	35.8 ± 5.9	23.8 ± 4.6

^a^Theoretical patterns based on changing 25% or 50% of red cones in the native control patterns to rod identity.

Additional experiments were performed with embryos treated at 36 hpf and analyzed later in development (75 hpf). In these experiments, the densities and expression levels of cells expressing rod opsin or rod transducin (*gnat1*) in RA-treated retinas were so elevated as to preclude measurement of rod density (Table 1 and data not shown). The densities of red and blue cones at 75 hpf were also significantly lower following RA treatment than in controls, with red cones affected to a greater extent than blue cones (Table 1).

**Figure 3:**
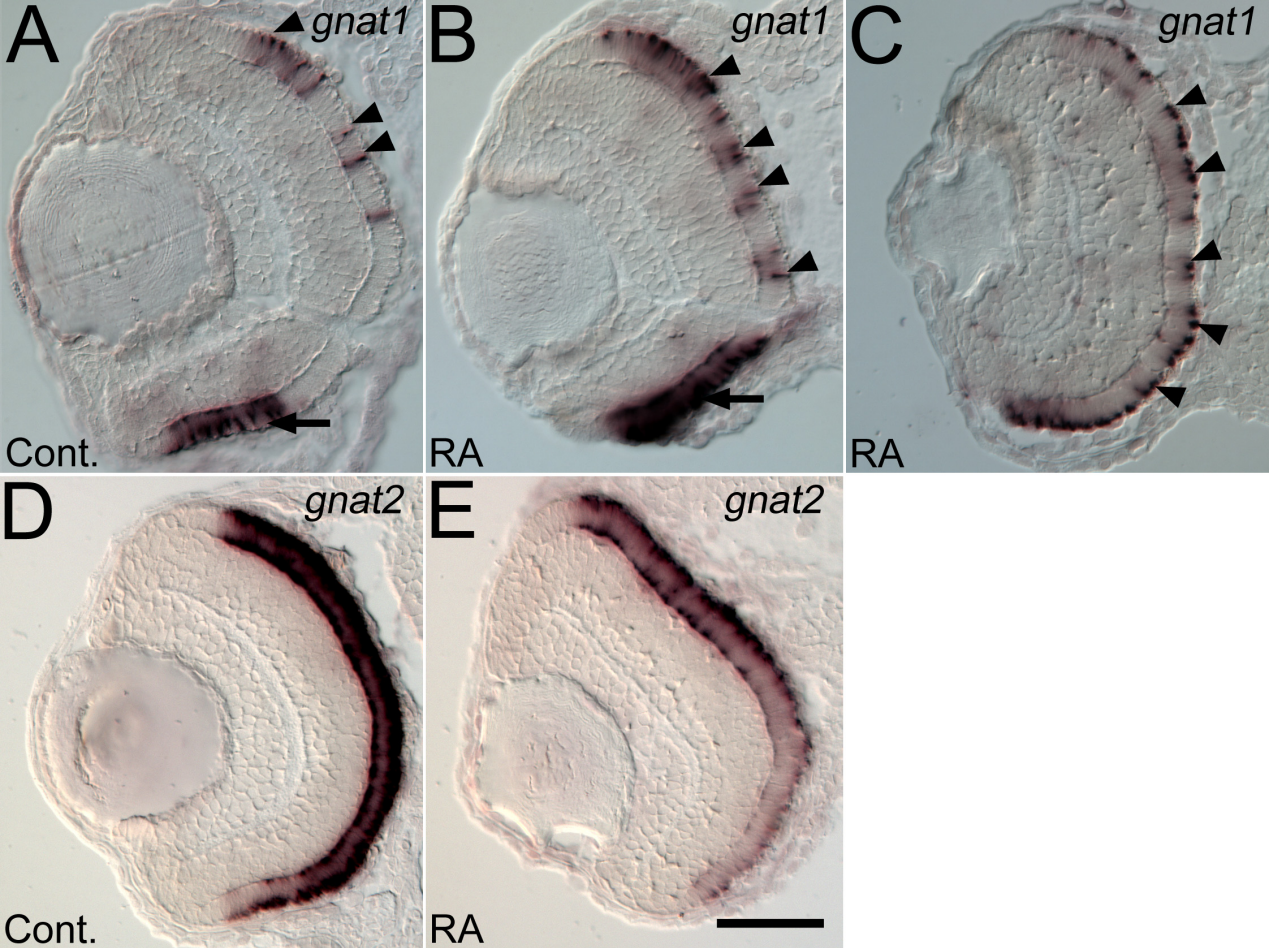
**Expression of rod and cone transducin genes is altered by retinoic treatment beginning at the time of retinal neurogenesis**. Embryos were treated with DMSO (Cont.; A, D), or 0.3 μM RA (B, C, E) at 36 hpf, and at 72 hpf were hybridized as 4 μm cryosections with probes corresponding to rod transducin (*gnat1*; A, B, C), or cone transducin (*gnat2*; D, E). Dorsal is up in all panels. In control embryos *gnat1 *is expressed in the ventral patch of rod photoreceptors (arrow in A) and in dispersed cells on the dorsal side of the retina (arrowheads in A). In some RA-treated embryos *gnat1 *expression is more intense in the ventral patch (arrow in B) and in all RA-treated embryos *gnat1 *expression is found in more cells in the dorsal and central retina (arrowheads in B and C). The section in panel C is positioned more peripherally in the retina compared to sections in other panels. Control embryos show an even distribution of *gnat2 *expression (D), which is reduced in intensity in RA-treated embryos (E). Bar = 50 μm.

Expression of rod transducin, *gnat1*, and the gene encoding cone transducin (*gnat2; *expressed in all cone photoreceptors [45]) was analyzed on tissue sections in embryos treated at 36 hpf and fixed at 72 hpf. Sustained RA treatment beginning at 36 hpf caused an apparent increase in the intensity of *gnat1 *expression and the appearance of additional *gnat1*^+ ^cells (Figure 3A, B, C, arrowheads). Cells were counted on sections hybridized to *gnat1*, and a significant increase in the number of *gnat1^+ ^*cells was observed for RA-treated embryos. Control embryos (n = 4) had on average 16 ± 5 *gnat1^+ ^*cells per section (15 sections), whereas RA-treated embryos had a significantly higher average 27 ± 6 *gnat1^+ ^*cells per section (24 sections, p < < 0.001, Student's T-Test). In contrast, RA treatment caused a decrease in the intensity of *gnat2 *expression in the retina (Figure 3D, E). The diffuse staining pattern of the *gnat2 *hybridization on tissue sections and on whole mounted specimens precluded a quantitative assessment. Collectively these findings indicate an increase in the number of cells expressing rod photoreceptor genes, and a decrease in cells expressing cone photoreceptor genes following sustained RA treatment beginning in early retinal neurogenesis.

### Sustained RA treatment beginning at the time of early retinal neurogenesis alters rod and cone patterns

The RA-dependent changes in the number of rod and cone photoreceptors, and the unusual two dimensional mosaic patterns thus formed, together suggested that the RA treatment at 36 hpf may have influenced the pool of progenitor cells competent to produce photoreceptors, to favor a production of rods over cones. A possible explanation is that the postmitotic daughters of RA-exposed progenitor cells originally positioned to differentiate as cones - that is, cone precursors - switched to the rod fate. A comparable situation has been observed in the zebrafish *tbx2 *mutant. In *tbx2-/- *retinas, rod photoreceptor production is favored over the production of UV cones, and the additional rods assume positions of the missing UV cones [13]. Furthermore, in the zebrafish, rod progenitors are molecularly similar to cone progenitors [11] and cone precursor cells are likely born close to their final positions in the two dimensional photoreceptor mosaic [46]. Thus it is reasonable to predict that in RA-treated embryos the resulting two dimensional patterns of rod photoreceptors will assume some of the spatial features of the cone photoreceptor mosaic. This prediction was tested by using two-dimensional pattern analysis techniques to evaluate and compare objectively the spatial features of the rod and cone mosaics in treated and control retinas [31].

#### Local Pattern of Cones

The mean Nearest Neighbor Distances (NNDs) were determined and Conformity Ratios (CRs; Mean NND/S.D.) calculated for native red cone, blue cone, and UV cone patterns. The resulting CRs were high (Additional File 1, Table S1), consistent with the presence of significant local pattern regularity for each cone type [47] suggesting that photoreceptor patterning mechanisms are operational at the time of, or earlier than initial photoreceptor differentiation [46,48].

**Figure 4:**
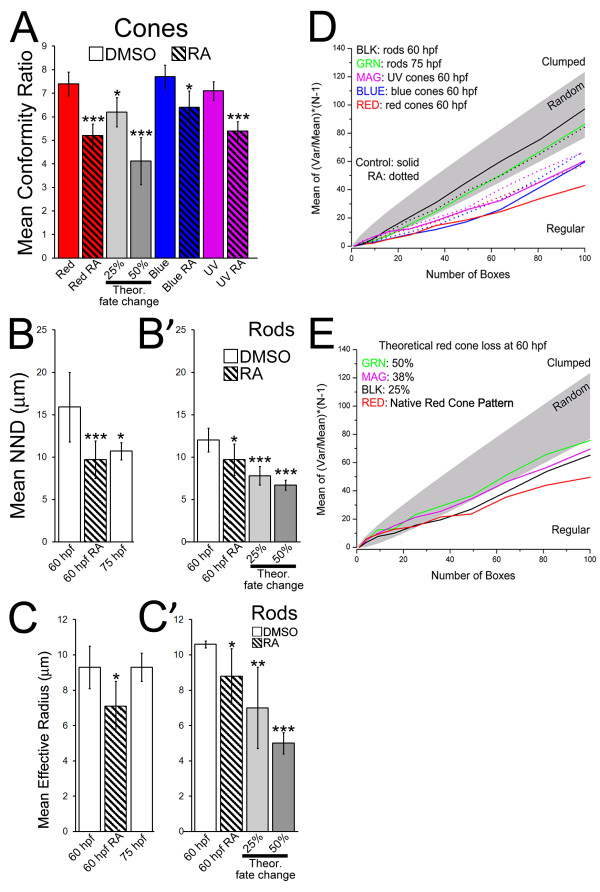
**Retinoic acid treatment results in significant differences in the two-dimensional pattern of photoreceptors**. (A) Conformity Ratio (CR) analysis of red, blue, and UV cones at 60 hpf. RA treatment caused significantly reduced regularity in spacing. Theoretical fate changes of 25% and 50% (see Methods) also resulted in significantly reduced CRs. (B) Nearest Neighbor Distance analysis (NND) of rods in singly-hybridized retinas at 60 hpf. RA treatment resulted in a significant decrease in NNDs (Additional File 1, Table S1; p < 0.001). (B') Autocorrelative NND analysis of rod patterns from doubly-hybridized retinas (rod and red cone opsin). Theoretical fate changes (25%, 50% of red cones to rods) also resulted in significantly reduced mean NNDs. (C) Density Recovery Profile analysis (DRP) of rods in singly hybridized retinas at 60 hpf. RA caused a significantly smaller Effective Radius (R_eff_; Additional File 1, Table S1; p < 0.05); this change does not take place in untreated retinas sampled later, at 75 hpf (C') Autocorrelative DRP analysis for rod patterns from doubly-hybridized retinas (rod and red cone opsin). Theoretical fate changes also resulted in significantly reduced mean R_eff_s. (D) Quadrat analysis compares long-range pattern of rods and cones in control (solid lines) and RA-treated (dotted lines) embryos. With RA, long-range pattern of rods becomes more regular while those of all cone types become more random. (E) Quadrat analysis of theoretical red cone patterns reveals decreasing pattern regularity (compare red line to green) as a consequence of an increasing percentage of loss from the red cone mosaic.

The analysis utilized an approach from our previous study [31] by initially evaluating the capacity of different pattern analysis methods to detect a fate switch. Multiple theoretical cone patterns exhibiting 'fate changes' were generated by randomly deleting 25%, or 50% of cells from samples of native red cone mosaics derived from 60 hpf and 75 hpf untreated embryos. In the resulting theoretical datasets, correspondingly greater mean cone NNDs were obtained (Additional File 1, Table S2). CRs calculated from these NNDs were significantly decreased in the cone patterns in which fate changes had been imposed (Figure 4A; Additional File 1, Table S2), reliably reflecting the decreased regularity of the altered theoretical cone patterns. We also performed Density Recovery Profile (DRP) analysis on theoretical cone patterns, but the effective radii (R_eff_) showed no significant differences that reflected theoretical fate change (Additional File 1, Table S1). This suggests that CRs derived from NND analysis of cone patterns represent the most appropriate metrics for objectively detecting, and statistically evaluating, potential fate switches cause by exposure to exogenous RA in the experiments.

NND analysis was applied to cone patterns obtained from RA-treated embryos. The resultant CRs of these patterns were significantly lower (i.e., the cone patterns were less regular) in RA-treated vs. control retinas for red, blue, and UV cones analyzed at 60 or 75 hpf (Figure 4A; Additional File 1, Table S1). The reduced regularity of the local pattern cones in RA-treated embryos, as measured by CR values, was similar to that attained for theoretical datasets to which a 25% fate change had been applied (Figure 4A; Additional File 1, Table S2). The NND analyses are thus consistent with at least 25% of the cones adopting a different fate in the RA-treated embryos.

#### Local Pattern of Rods

NND analysis of native rod patterns in embryonic retina generated high CRs (Additional File 1, Table S1); however, we could not definitively determine if this indicated significant local pattern regularity due to the small number of rod cells in each sample [47]. Theoretical rod patterns were generated that exhibited 'fate changes,' by randomly switching the identity of 25% or 50% of red cones to rods in samples of native red cone and rod mosaics derived from 60 hpf untreated embryos. For this analysis, double-labeled material (Figure 2) was used so that any extant spatial relationships between rods and red cones at this developmental time would be incorporated, and each cell population was then subjected to autocorrelative pattern analyses. In these theoretical datasets, the imposed fate changes caused significant decreases in rod NNDs (Figure 4B'; Additional File 1, Table S3). The corresponding CRs did not show consistent, significant differences as a function of theoretical fate change (Additional File 1, Table S4), suggesting that the CR is not as useful for this analysis as the NND. In contrast, DRP analysis on theoretical rod patterns did indicate increasingly significant changes in the R_eff _values as a function of theoretical fate change percentage (Figure 4C and 4C'; Additional File 1, Table S5).

We therefore applied NND and DRP analyses to the rod mosaics of RA-treated retinas, including mosaics from both single and double-labeled retinas (Figure 4B, C; Additional File 1, Tables S1 and Tables S3 to S5). These rod mosaics displayed significantly decreased mean NND values in RA-treated retinas that approached, but did not quite match, the NNDs of cones (Additional File 1, Tables S1 and S3). When compared to the theoretical data, this result was consistent with the possibility of up to 25% of the red cones adopting a rod identity, resulting in a more compact "cone-like" local spacing of the rod mosaic (Figure 2B). This result, however, is also consistent with RA causing an accelerated differentiation of existing postmitotic rod precursors into the retina, because the NNDs of rods in RA-treated retinas at 60 hpf matched those of rods in untreated retinas sampled later, at 75 hpf (Figure 4B; Additional File 1, Table S1).

The DRP analysis was used to distinguish these two alternatives. The DRP analysis indicated a significant, RA-induced reduction in the mean R_eff _value for rods (i.e., rods were atypically proximal to other rods across the retinal sheet (Figure 4C; Additional File 1, Table S1). This result is consistent with a 25% fate change (Figure 4C and 4C'; Additional File 1, Tables S2 and S5). However, it is not consistent with accelerated differentiation of postmitotic rod precursors. In control retinas the R_eff _for rods was not significantly different between the 60 and 75 hpf evaluation times (Figure 4C; Additional File 1, Tables S2 and S3). Instead RA treatment during retinal neurogenesis results in altered rod patterns with significantly decreased local spacing that is distinct from the rod patterns observed in older embryos. Accelerated differentiation of existing, 'pre-positioned' rod precursors into the retina therefore does not explain the RA-induced increase in rod density.

#### Cross-Correlative Analysis

Samples from retinas labeled simultaneously for rod opsin and red cone opsin mRNAs also were subjected to cross-correlative pattern analyses to examine changes in red cone-to-rod and rod-to-red cone spatial relationships. Interestingly, the cross-correlative, red cone-to-rod NND analysis generated high CR values indicating a regular pattern (Additional File 1, Table S4). For cross-correlative samples, this in turn indicates the existence, at these developmental times, of a predictable, non-random spacing relationship between red cones and rods in native retina.

Cross-correlative NND analysis was also somewhat predictive of fate changes when applied to theoretical datasets in which red cones were switched to the rod fate. Changes in the mean NND and corresponding CR values from red cone-to-rod and rod-to-red cone comparisons were significantly different from controls when between 25% to 50% of the red cones assume rod identity (Additional File 1, Table S3). NND analysis of experimental material revealed that RA caused a significant decrease in NND between red cones and rods (cross-correlative). These values were consistent with 20-25% fate changes (Additional File 1, Table S4).

#### Global Patterns of Cones and Rods

To supplement the NND and DRP analyses, which are predominantly measures of local pattern characteristics, quadrat analysis, a measure of global (long-range) pattern that encompasses, in general, the 'repeatability' of local pattern motifs across two-dimensional space [49,50], was utilized. Quadrat analysis was applied to patterns derived from singly-labeled retinas.

Figure 4D shows the averaged quadrat results for all patterns analyzed, by cell type and treatment. Native rod patterns (60 and 75 hpf) were statistically equivalent to a random pattern, whereas all cone patterns (60 and 75 hpf, all cone types) were statistically regular (Figure 4D). Quadrat analysis of the theoretical fate change datasets revealed significant decreases in long-range order of red cones that accurately reflected the extent of theoretical fate change (Figure 4E). The cone patterns in RA-treated retinas were significantly less regular on a global scale than the corresponding controls (Figure 4D), consistent with the introduction of gaps, due to significant cone loss, within the typically highly regular long-range pattern of the zebrafish cone mosaic [51]. These changes in cone pattern corresponded to fate changes of approximately 25% (compare to Figure 4E), and are similar to those independently estimated from the NND analyses and the measurement of red cone density (see above).

The long-range patterns of rods in RA-treated retinas were also significantly more regular than those of their control counterparts (Figure 4D). While this particular analysis will not permit the estimation of a corresponding magnitude of fate change, the result nevertheless indicates that the rods in RA-treated embryos, although atypically proximal on local scales (see above), tend over larger scales to occupy positions normally devoid of rods. The effect is the introduction of a certain degree of long-range spatial regularity. The quantitative analyses of spatial patterning thus provide support for the hypothesis that RA exposure during retinal neurogenesis may function as a component of molecular mechanisms controlling rod and cone neurogenesis. In this context prolonged RA treatment causes some "proto-cones" to switch to the rod phenotype.

### Sustained RA treatment beginning in early retinal neurogenesis does not result in 'opsin switching'

**Figure 5:**
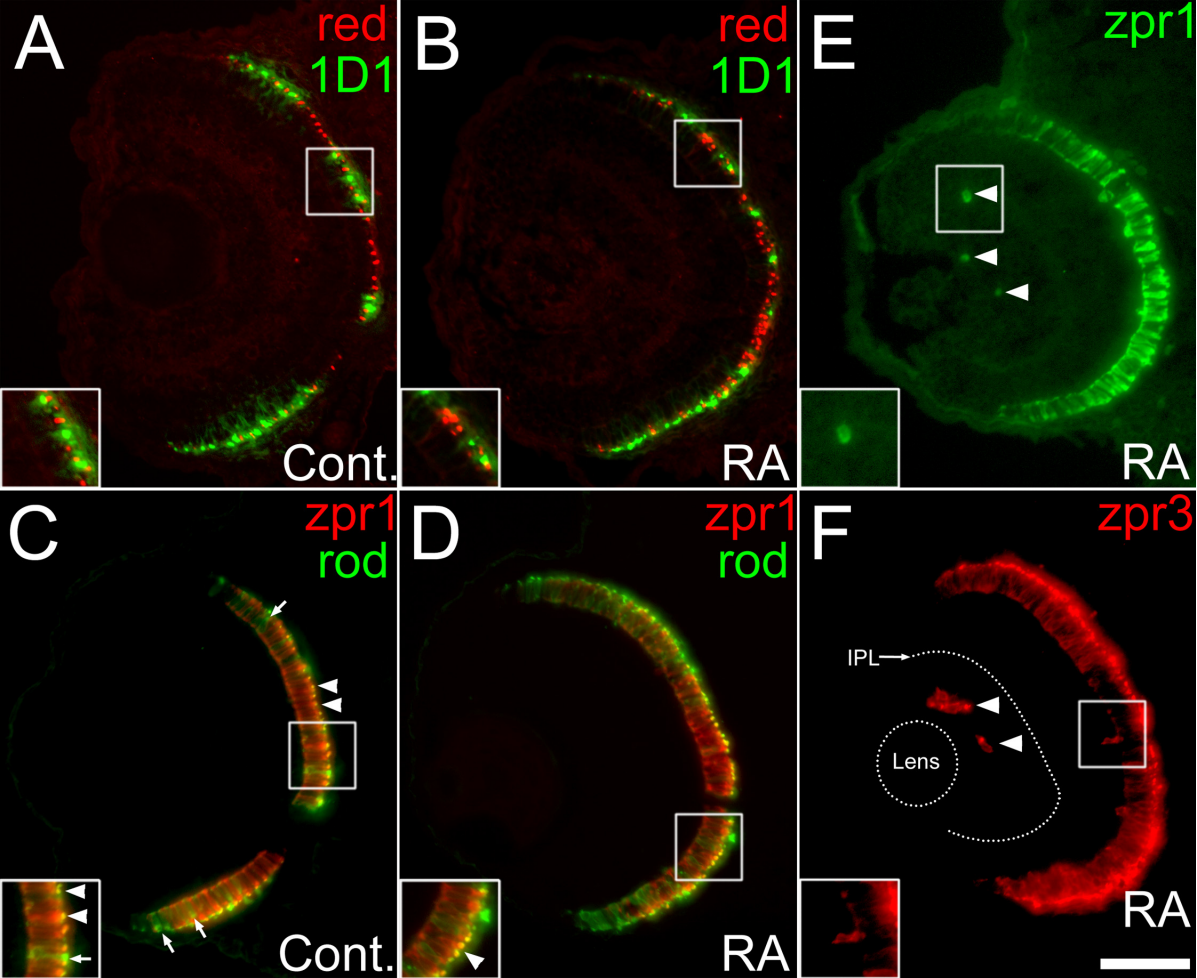
**Retinoic acid treatment during retinal neurogenesis does not result in photoreceptors of mixed phenotype**. Control embryos were treated with DMSO (A, C), or 0.3 μM RA (B, D to F) at 36 hpf, and at 60 hpf were processed as 5 μm cryosections for indirect immunofluorescence with rod- and cone-directed markers. Boxed regions in each panel appear at higher magnification in the insets. (A, B) Embryos were stained with a polyclonal anti-red opsin antibody (red color, stains red opsin), and monoclonal antibody 1D1 (green color; specific for rod opsin). Sections from control (A) or from RA-treated (B) embryos showed no evidence of colabeling. (C, D) Embryos were stained with zpr1 (red color, labeling red and green-sensitive cones) and a polyclonal anti-rod opsin antibody (green color, stains rods as well as green cones). In control embryos (C), there are singly- as well as doubly-labeled cells, indicating the likely presence of red cones (red color), green cones (yellow color; arrowheads), and rods (green color, arrows). (D) In embryos treated with RA, there is reduced staining of red cones and green cones and more intense staining for rods. (E, F). In an RA-treated embryo, cells positive for zpr1 (E) and the rod/green cone marker zpr3 (F) are found in ectopic locations (arrowheads). IPL, inner plexiform layer. Bar = 50 μm.

The quantitative changes to rod and cone patterns induced by RA treatment beginning at 36 hpf suggested that some of the cells otherwise fated and positioned to become cones instead became rods. An alternative explanation is that cone vs. rod fate was not affected, but cones expressed rod opsin instead of the appropriate cone opsin. Arguing against this alternative are the increase in *gnat1*-expressing cells and the decrease in *gnat2 *expression (Figure 3), and the apparent lack of colabeling in the dual in situ experiments (Figure 2). However, as a further test of this alternative 'opsin switching' hypothesis explicitly in RA-treated retinas, mis-expression of rod opsin by (otherwise) cone photoreceptors was evaluated with an antibody that is selective for rod opsin (1D1; [52]), together with a polyclonal antibody to red cone opsin (see Methods). In sections derived from both control and experimental retinas no co-labeled cells were evident (Figure 5A and 5B).

A different set of markers was also used to test the same hypothesis. A polyclonal rod opsin antibody was applied together with zpr1, the latter an immunological marker for red- and green-sensitive double cones (Figure 5C and 5D). The rod opsin antibody labels apical processes of both rods and green opsin-expressing cones, due to the similar primary structures of rod and green cone opsin (T. Vihtelic, personal communication). Therefore, control embryos displayed a combination of rod opsin^+ ^cells (rods), zpr1^+^/rod opsin^- ^cells (red cones), and colabeled cells (green cones; Figure 5C, arrowheads). If cones in RA-treated retinas mis-expressed rod opsin, an increase in co-labeled cells following RA treatment would be expected. No such increase in colabeling for zpr1 and rod opsin was observed (Figure 5D). Instead, rod opsin labeling appeared more intense and widespread. These immunolabeling experiments support the hypothesis of an RA-induced cone-to-rod fate change, and provide no empirical evidence for 'opsin switching.'

### RA treatment causes an increase in ectopic expression of photoreceptor markers

It was noted during the immunolabeling analyses that cells labeled with the red and green cone cell marker zpr1 and the rod cell marker zpr3, were observed in locations other than the ONL (Figure 5E, F, arrowheads). Such cells, which were present in both control and experimental embryos, were previously documented in wild-type zebrafish as ectopic photoreceptors located within the INL and the GCL [53]. Analysis of RA-treated and control retinas indicated an RA-dependent increase in the probability of encountering ectopic photoreceptors (i.e. cells that were zpr1^+ ^or zpr3^+ ^cells in the INL (Table 3). As a further analysis, the number of ectopic cells was counted in three zones in the retina: the GCL, the proximal to middle part of the INL, and the distal INL (located adjacent to the ONL). In RA-treated retinas, there was a significant increase in the number of zpr1^+ ^and zpr3^+ ^cells (cones and rods, respectively) found in the distal INL (Table 3, see Figure 5F, inset). A small but significant increase in number of ectopic zpr3^+ ^cells (rods) was counted in the proximal to mid-INL region. No significant change in the number of ectopic photoreceptors in the GCL was observed. The presence of ectopic zpr3^+ ^cells after exposure to RA in embryonic zebrafish also was observed, but not quantified, in a previous study [44]. The results in the present study suggest that the effects of sustained high RA signaling upon photoreceptor production and placement are not restricted to the ONL.

**Table 3:** Presence of ectopic photoreceptors in control and RA-treated embryos

	% of embryos with ectopic photoreceptors			
	**zpr1**^**+**^		**zpr3**^**+**^	
	Control	RA	Control	RA
**Found in GCL**	57%	86%	57%	86%
**Found in INL**	29%	100%^§^	14%	100%^§^

	**Average number of ectopic photoreceptors per eye**^**a**^			

	**zpr1**^**+**^		**zpr3**^**+**^	
	**Control**	**RA**	**Control**	**RA**

**GCL**	0.92 ± 1.55	2.31 ± 4.79	3 ± 3.64	4.14 ± 5.08
**INL (prox + mid)**	0.08 ± 0.28	0.15 ± 0.38	0	0.64 ± 0.93*
**INL (distal)**	0.15 ± 0.38	2.38 ± 2.26**	0.14 ± 0.53	1.86 ± 1.79**

^§ ^p < 0.05 (Fisher Exact Test).

^a ^ ± S.D.

*p < 0.05; **p < 0.01;***p < 0.001 (Student's t-test).

### Effects of early RA treatment on cones is not due to cell death in the ONL

**Figure 6:**
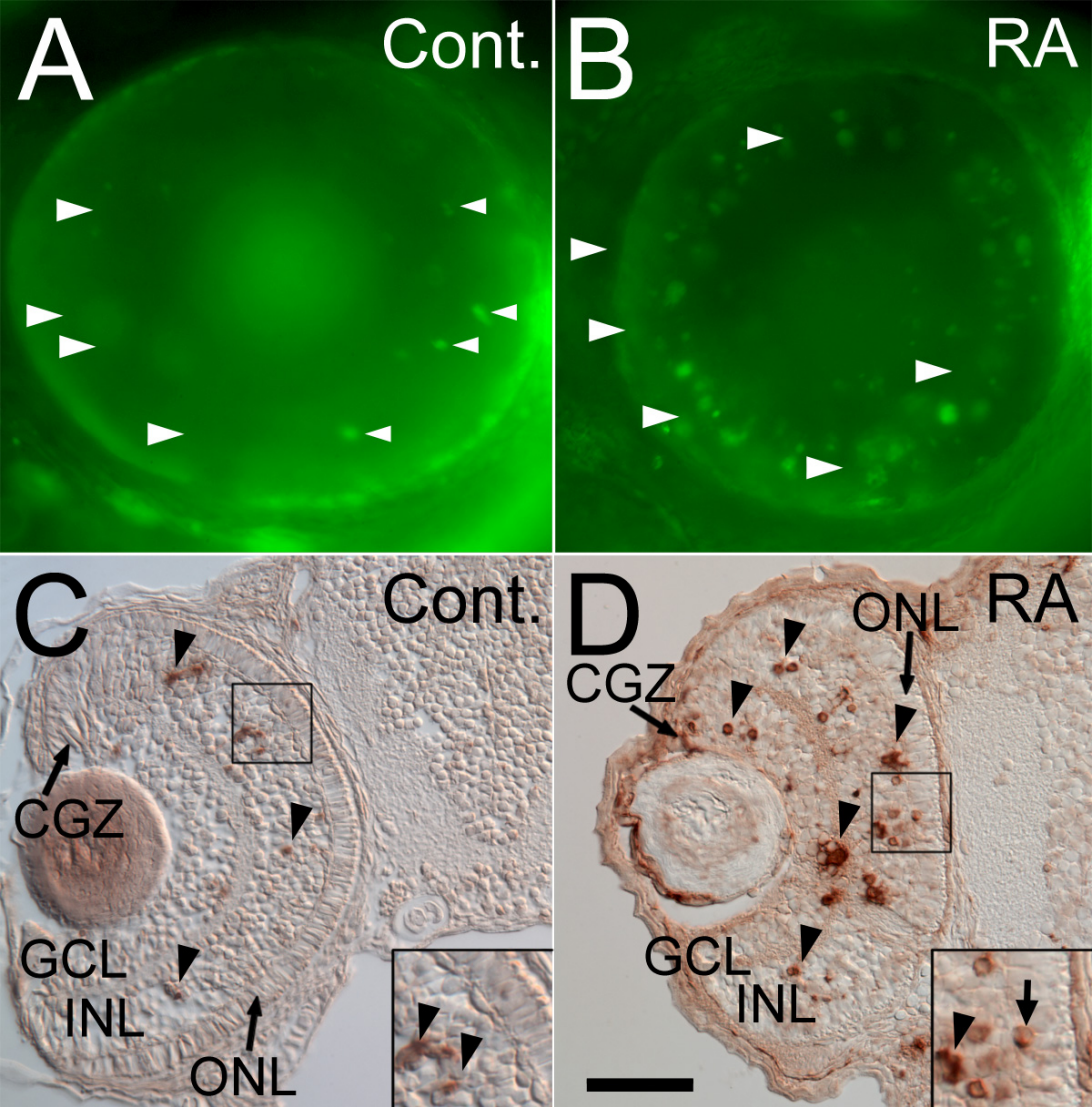
**Retinoic acid treatment during retinal neurogenesis results in a significant increase in retinal cell death**. Control embryos were treated with DMSO (A, C), or 0.3 μM RA (B, D) at 36 hpf, and either were stained live at 75 hpf with the cell death marker Acridine Orange and prepared as whole mounts for viewing (A, B), or were sectioned at 5 μm and processed for cell death detection with the TUNEL kit (C, D); dorsal is up in all panels. Control embryos showed very few dead/dying cells (arrowheads in A), and these were found predominantly in the inner nuclear layer (C), while those treated with RA (D) showed widespread cell death in the inner nuclear layer (INL), ganglion cell layer (GCL), and circumferential germinal zone (CGZ, D). However, little cell death was detected in the outer nuclear layer (ONL) (D). Bar = 50 μm for all panels.

An alternative explanation for red cones missing from the mosaic is that incipient red cones manifested cell death prior to terminal differentiation. This alternative was tested with cell death assays. Acridine orange staining of 75 hpf embryos treated with RA at 36 hpf (Figure 6B) labeled a larger number of dying cells within the eye as compared to controls (Figure 6A). The acridine orange^+ ^cells were located predominantly in the INL and in the lens. The TUNEL assay was used to determine with greater precision the number and distribution of dying cells subsequent to RA treatment (Table 4). RA treatment at 36 hpf resulted in an increased number of apoptotic cells in embryos assayed at 60 hpf (Figure 6C, D). RA-treated embryos (n = 4) had on average nearly four times as many TUNEL-positive cells per section compared with control embryos. Cell death was quantified for defined retinal locations and was again found to be significantly higher in RA-treated retinas, for all retinal regions evaluated (Table 4). However, the RA-induced increase in cell death in the ONL was low compared to the evident increase in the GCL, INL, and CGZ (the peripheral region of the retina displaying no overt indicators of lamination). These data were compared to the red cone densities presented above (Table 1). The number of TUNEL+ cells over the dimensions of the surface areas scored for cell death was approximately 0.00021 TUNEL^+ ^cells per μm^2 ^of the ONL. In contrast, the decrease in the average red cone density between control and RA-treated embryos was 0.005 cells per μm^2^, a difference that is ~24 times greater than the level of cell death. This suggests that cell death in the ONL is not a major factor affecting cone photoreceptor patterns in the RA-treated embryos.

**Table 4:** Effect of RA on cell death in different retinal regions

	Average number of TUNEL+ cells per section				
	ONL	INL	GCL	GZ	Total
Control (28 sections)	0	3.86	0.50	0.04	4.39
RA (22 sections)	0.43**	9.23***	2.64***	4.14***	16.41**

*p < 0.05; **p < 0.01; ***p < 0.001 (Student's t-test).

The potential role for cell death was tested further by repeating the RA treatment on embryos injected with an antisense morpholino (MO) directed against *p53 *[54], which has been shown to block apoptotic cell death. However, the p53-MO did not block RA-induced retinal cell death (data not shown), and so it was not possible to evaluate the effects of RA completely independently of cell death.

### Proliferating retinal progenitor cells are responsive to RA signaling

At the time of initiation of RA treatments (36 hpf), cells that will occupy the ONL are still proliferative, and by 48 hpf cells of the ONL have begun to exit the cell cycle and differentiate [1]. Previous studies have shown that endogenous RA signaling in the developing zebrafish retina exists in a strong ventral domain and a weaker dorsal domain [31,55]. The restricted size of these endogenous RA signaling domains suggests that cells outside these areas, including many of the proliferating retinal progenitors, may not possess the ability to respond directly to RA. This possibility was investigated by utilizing embryos from a zebrafish line carrying the RA signaling reporter transgene, RARE-YFP (RGnY; [55]).

**Figure 7:**
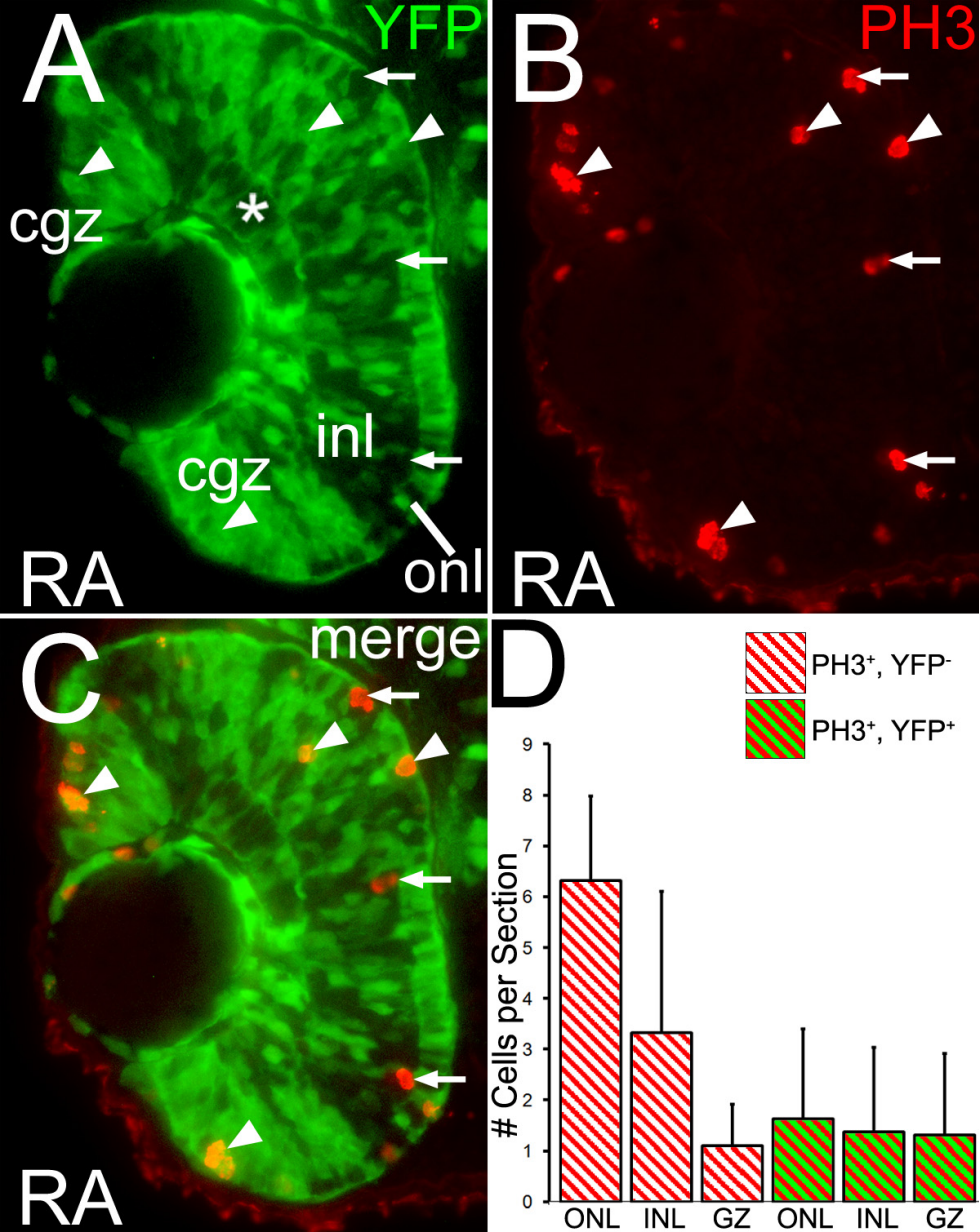
**Proliferating cells in the retina can activate a reporter gene in response to RA**. Embryos carrying the RARE-YFP transgene were treated with 0.3 μM RA at 36 hpf, and at 48 hpf were processed as 3 μm cryosections for indirect immunofluorescence with an anti-GFP antibody (green color; A) and a marker for mitotic cells, anti-phosphohistone H3 (red color; B); panel C shows the merged image. Dorsal is up. Asterisk indicates the ganglion cell layer (GCL). There are mitotic cells in the retina that are both negative (arrows) and positive (arrowheads) for YFP expression. (D) Numbers of singly-labeled (PH3^+^, YFP^-^) and doubly-labeled (PH3^+^, YFP^+^) mitotic cells were counted as a function of position in the retina in a sample of sections from RA-treated embryos. Cells counted in the developing ganglion cell layer were lumped together with those of the inner nuclear layer (INL). Single-labeled anti-PH3 cells are found largely in the outer nuclear layer (ONL) and INL. Few single-labeled anti-PH3 are seen in the GZ. Colabeled cells are found evenly distributed among these retinal regions. Bar = 50 μm.

Transgenic embryos exposed to RA from 36 to 48 hpf displayed widespread and intense YFP expression in the retina at 48 hpf (Figure 7A). To identify YFP^+ ^cells that are also mitotic, sections were co-labeled with an antibody to phosphohistone H3 (PH3), a marker for cells within M phase of the cell cycle. At 48 hpf, PH3^+ ^cells were observed that were colabeled (Figure 7C, arrowheads), and those that were not colabeled for YFP (Figure 7C, arrows), within the CGZ, the developing ONL, and the INL (Figure 7C). PH3^+ ^cells within the developing ONL likely correspond to photoreceptor precursors [1]; those within the INL may correspond to cells of the rod photoreceptor lineage [11]. Singly- and doubly-labeled cells in three different retina regions, the ONL, INL and CGZ, were counted to determine if there was a specific pattern to the labeled cells. For simplicity, due to its small size, the developing GCL was included in the INL zone. At 48 hpf the highest number of (PH3^+^, YFP^-) ^cells were found in the ONL, with half as many in the INL and few in the CGZ region (Figure 7D). In contrast, (PH3^+^, YFP^+^) cells were observed to be more evenly distributed among the three regions (Figure 7D). These data indicate that some proliferating cells within embryonic retina were directly responsive to RA signaling.

### Sustained RA signaling in retinal progenitor cells and postmitotic retinal cells is required for changes in photoreceptor ratios and pattern

To test the hypothesis that exogenous RA influences photoreceptor pattern via effects exclusively within retinal progenitor cells, we treated RARE-YFP transgenic embryos with a brief 'pulse' of 0.3 μM RA, from 36 to 39 hpf. In these embryos, increased RA signaling was verified as transient, as the RARE-YFP transgene was no longer upregulated at 49 hpf (Additional File 2, Figure S1A, B), a time when the first photoreceptors withdraw from the cell cycle and begin to differentiate [1,3,56]. In a parallel experiment, embryos treated with a similar pulse of RA from 36 to 39 hpf displayed qualitatively normal rod and cone photoreceptor patterns when examined at 60 hpf (Additional File 2, Figure S1C, D, and data not shown). These results suggest that increased RA signaling in retinal progenitors, prior to terminal mitosis, is not sufficient to modify photoreceptor fate. The results of [31] also excluded a role for RA exposure at later developmental times in regulating photoreceptor fate. Collectively these studies suggest that prolonged, increased RA signaling in retinal progenitors and in their postmitotic photoreceptor precursors, is required to favor the production of rod photoreceptors over that of cones.

**Figure 8:**
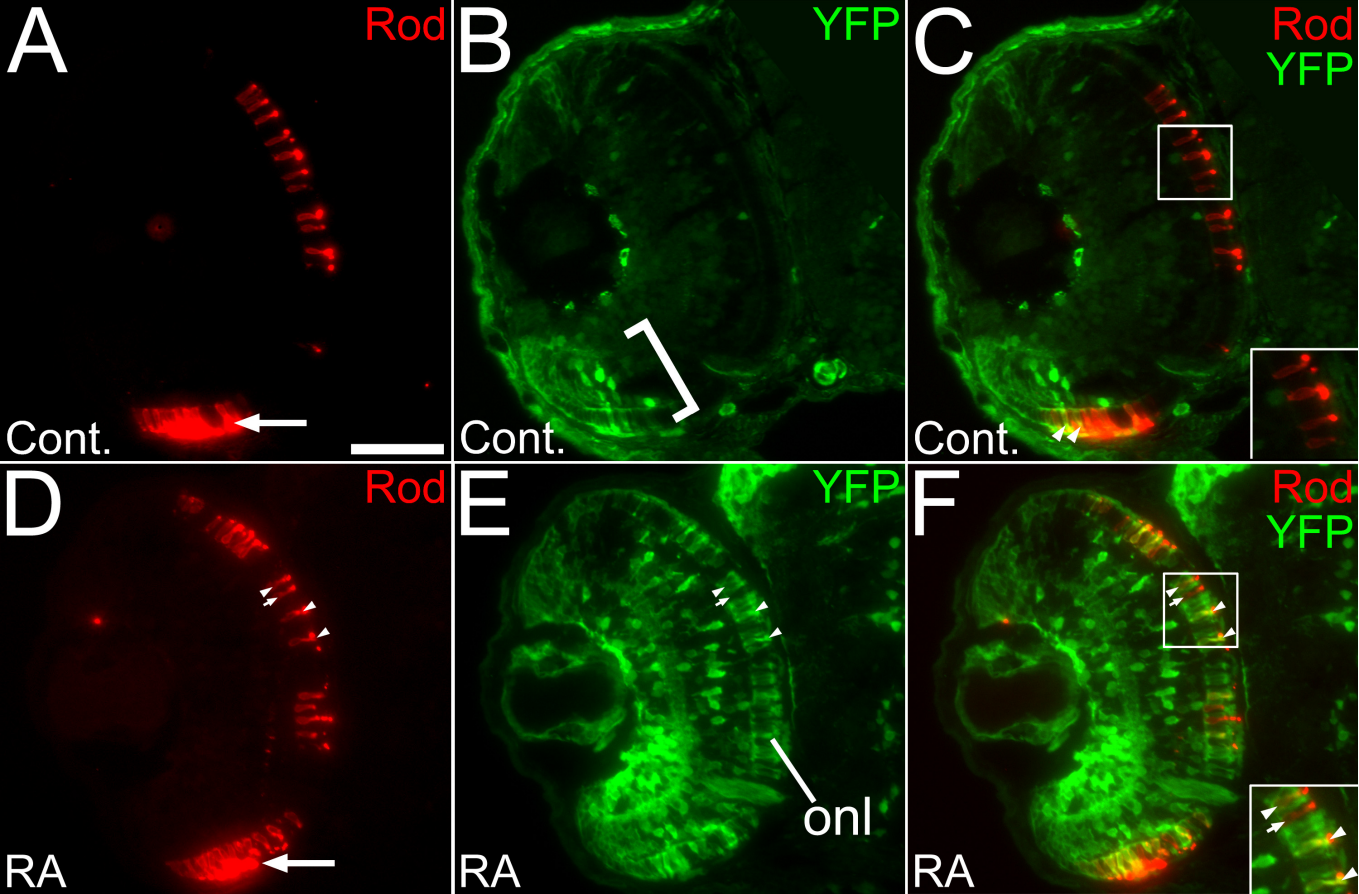
**Retinoic acid signaling within a subpopulation of rod photoreceptors in response to prolonged retinoic acid treatment**. (A to C) Embryos carrying the RARE-YFP transgene were treated with DMSO (A to C) or 0.3 μM RA (D to F) from 36 to 60 hpf, and were processed as 4 um cryosections for indirect immunofluorescence with an anti-GFP antibody (green) and antibody to rod opsin (1D1; red). (A) Rod photoreceptors are found densely populated in a ventral patch (arrow) and more widely distributed in the dorsal retina. (B) In control embryos, endogenous reporter expression is limited to cells of the ventral retina, but found in all retinal layers in that region (bracket). (C) Merged panel from A and B showing some colocalization of YFP and rod opsin (arrowheads) in the ventral retina, and none in the dorsal retina (inset). (D) Rod photoreceptors in a retina from an embryo treated with RA. Arrow indicates the ventral retina. (E) In experimental embryos the RA treatment leads to widespread expression of YFP, including the ONL. (F) Merged panel from D and E, showing rods in the dorsal retina colocalized with YFP (arrowheads) as well as those not expressing YFP (arrow). Bar = 50 μm.

To characterize the photoreceptors engaged in sustained, increased RA signaling, we treated transgenic RARE-YFP embryos with 0.3 μM RA, from 36 hpf to 60 hpf, a treatment that results in altered rod and cone patterns (Figures 1 and 4). We stained cryosections with antibodies directed against the YFP transgene, together with the 1D1 antibody, which detects rod opsin [52]. We observed YFP+/1D1+ rods, as well as YFP-/1D1+ rods (Figure 8). The proportion of rods in RA-treated embryos that were also YFP+ (half of all rods; Table 5; Figure 8) slightly exceeded the proportional increase in rods in RA-treated embryos as compared to DMSO-treated embryos (one third of all rods; Table 5). It is therefore possible that many of the rods engaged in sustained, increased RA signaling correspond to 'new' rods diverted from alternative fates. However, a small number of cells were also observed that colabeled with YFP and the cone marker zpr-1 (one out of every 10; Additional File 3, Figure S2), arguing against an exclusively cell-autonomous effect of increased RA signaling.

**Table 5:** Rod photoreceptors engaged in RA signaling following prolonged RA exposure

	Average number of rod photoreceptors per retina that are:		
	YFP-	YFP+	Total rods
DMSO (n = 4)	102 ± 12.7	none	102 ± 12.7
RA (n = 4)	75.3 ± 20.5	75.3 ± 8	152 ± 21.8*

*p < 0.05 (Student's t-test, vs. DMSO).

### Multiple differentiating cell types in the retina can engage in RA signaling

**Figure 9:**
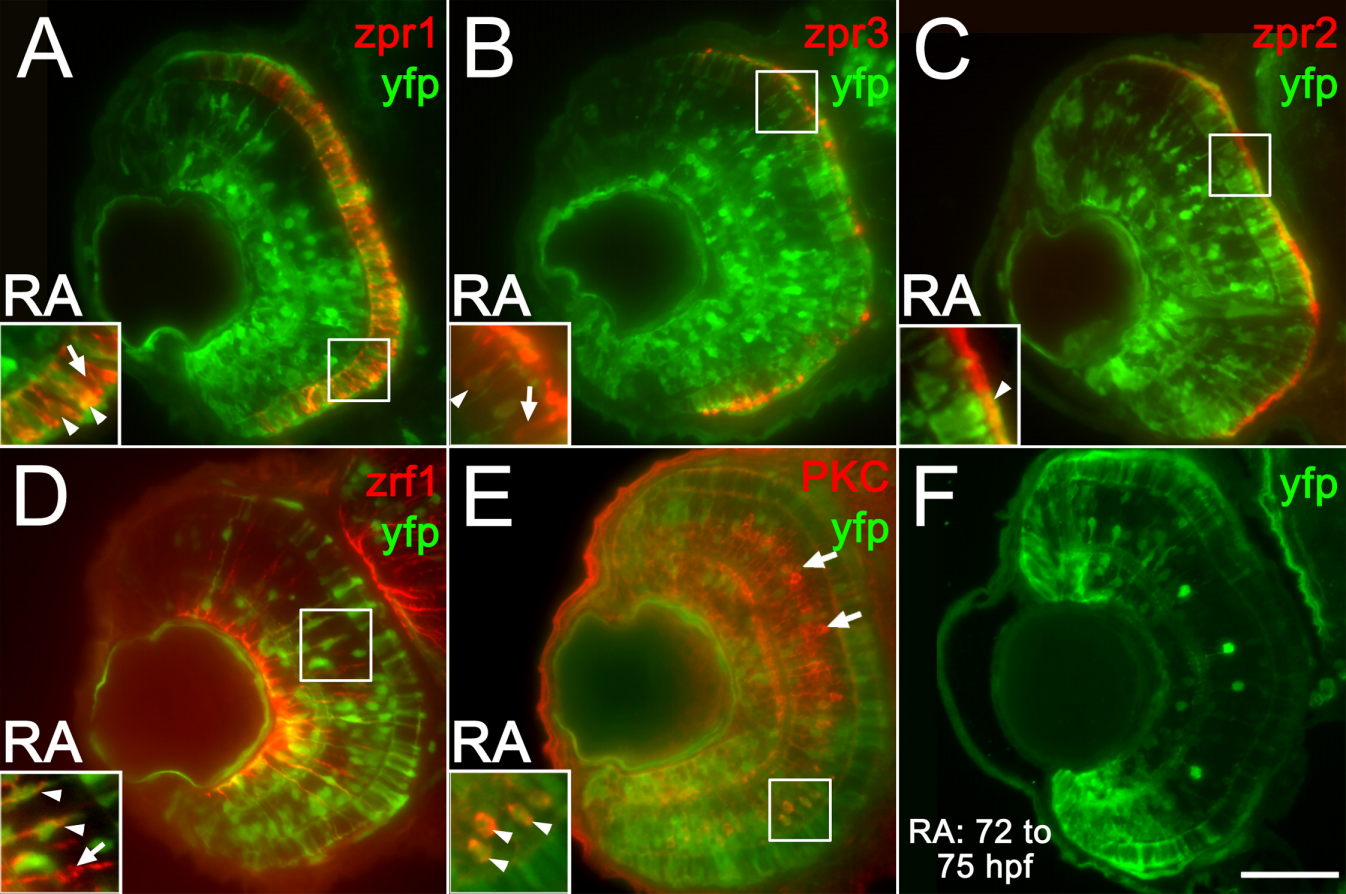
**Multiple retinal cell types engage in retinoic acid signaling in response to retinoic acid treatment during retinal differentiation**. (A to E) Embryos carrying the RARE-YFP transgene were treated with 0.3 μM RA at 48 hpf, and at 75 hpf were processed as 5 μm cryosections for indirect immunofluorescence with an anti-GFP antibody (green color in all panels) and the following markers (red color in all panels): zpr1 (stains red and green-sensitive cones; (A), zpr3 (stains rods and green cones; (B), zpr2 (stains RPE; C), zrf1 (stains Müller glia; D), and anti-PKC (stains rod bipolar cells; E). Dorsal is up in all panels; boxed regions in each panel appear at higher magnification in the insets. All types of retinal cells examined are capable of responding directly to RA by activating the RARE-YFP transgene (doubly-labeled cells in each panel; arrowheads; colabeling within the limit of resolution of our objective lens = 1.4 μm), although not all cells of each type respond (singly-labeled cells; arrows). (F) Embryos carrying the RARE-YFP transgene were treated with 0.3 μM RA at 72 hpf, and were fixed at 75 hpf as 5 μm cryosections for indirect immunofluorescence with the anti-GFP antibody. Widespread transgene expression indicates rapid response to RA. Bar = 50 μm.

Our earlier study demonstrated targeted roles for RA signaling during photoreceptor differentiation [31]. To determine if differentiated retinal cell types are also directly responsive to RA, specifically over a later RA treatment period, RARE-YFP embryos were exposed to RA at 48 hpf, a time at which all retinal cell types are extant. This exposure produced, at 75 hpf, widespread YFP expression across the retina, in all retinal layers (Figure 9A-E). The identity of RA-responsive cell types was determined by processing sections from treated embryos with an antibody against YFP and a series of additional cell markers. Labeling with a marker for red/green double cone pairs (zpr1) revealed many YFP^+ ^cones in the ONL (Figure 9A, arrowheads). Similarly, the use of a marker for rods (zpr3) revealed rods positive for YFP^+ ^(Figure 9B, arrowheads). However, not all photoreceptors were YFP^+^, suggesting that not all rods and cones can respond directly to RA during this later treatment period (Figure 9A, B, arrows). A similar situation was observed for cells of the retinal pigmented epithelium (RPE), identified using the marker zpr2: some RPE cells were YFP^+ ^(Figure 9C, arrowhead in inset), others were not. Using a marker for Müller glia (zrf1), multiple examples of co-labeled cells were observed (Figure 9D, arrowheads in inset), as well as many examples of Müller glia not responding directly to RA (Figure 9D, arrow in inset). Experiments with a marker for rod bipolar cells, anti-PKC, similarly revealed that some but not all rod bipolar cells are capable of responding directly to RA (Figure 9E). The signaling response is rapid, as the increased expression of YFP in response to RA treatment at 48 hpf was seen as early as 56 hpf (data not shown). A similarly strong, though less widespread, response to RA also occurred when embryos were treated at 72 hpf and examined at 75 hpf (Figure 9F). This brief exposure also revealed strong RA signaling within a small subpopulation of INL cells (Figure 9F). These results suggest complex temporal dynamics associated with the response of retinal cells to RA and demonstrate that multiple, differentiated cell types in the developing retina are responsive, perhaps in a cell-autonomous manner, to exogenous RA.

### RA receptors are expressed in late retinal progenitors and in developing photoreceptors

**Figure 10:**
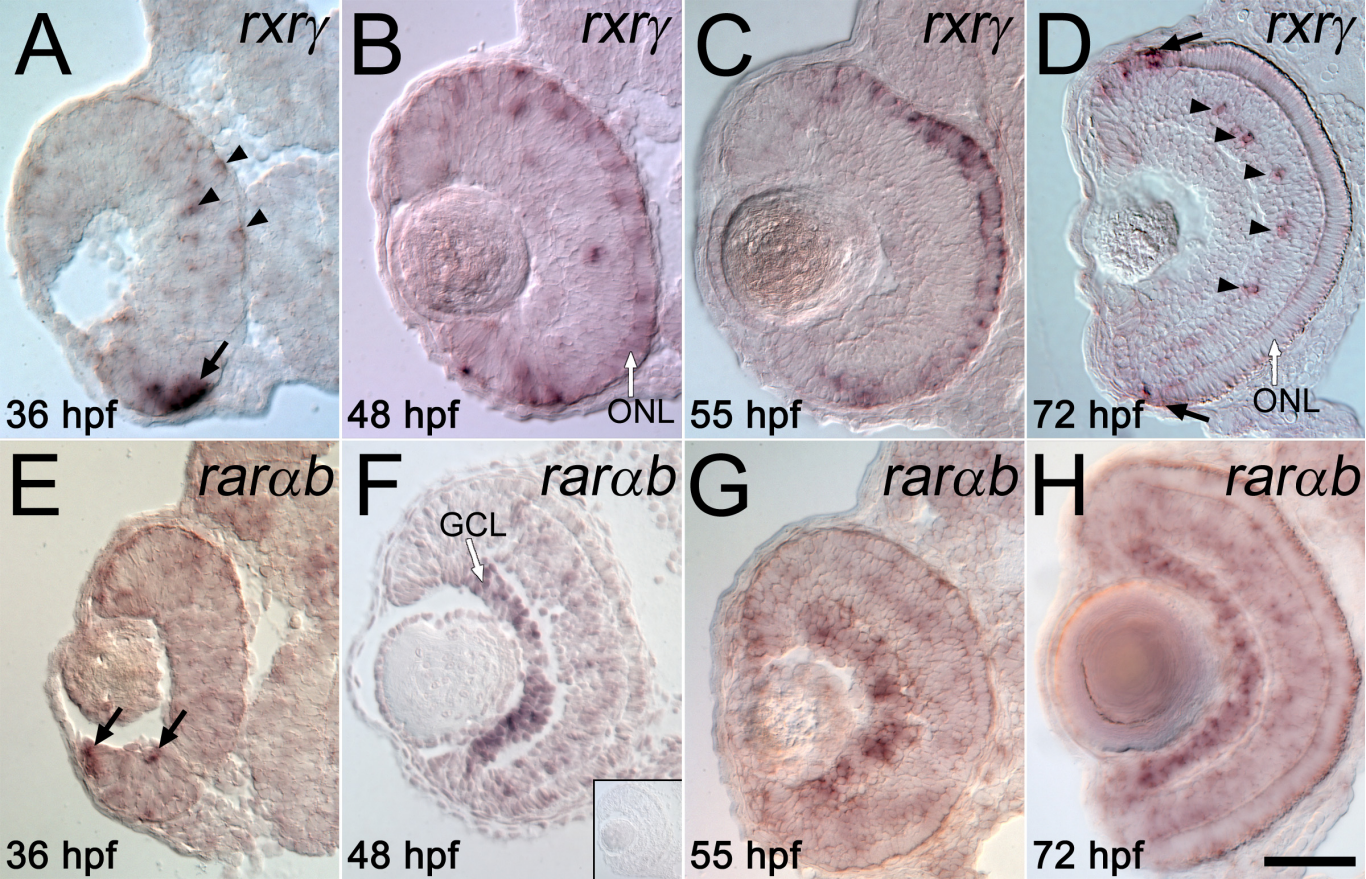
**Specific retinoic acid receptors are expressed in the embryonic zebrafish retina**. Wild-type, untreated embryos were fixed at 36 hpf (A, E), 48 hpf (B, F), 55 hpf (C, G), or 72 hpf (D, H), and were hybridized as 3 μm cryosections with probes corresponding to RXRγ (A-D) or RARαb (E-H); dorsal is up in all panels. (A to D) In younger embryos, RXRγ is expressed at the outer edge of ventral retina (arrow in A), and in scattered cells in inner and outer retina (arrowheads in A). In older embryos this expression becomes restricted to the emerging outer nuclear layer (ONL, B and C), and then to the most peripheral cells of the outer nuclear layer (black arrows in D) and periodically distributed cells of the inner nuclear layer (arrowheads in D). E.-H. In younger embryos, RARαb is expressed at the inner edge of ventral retina (arrows in E), and in older embryos shows widespread expression throughout the retina, with strongest hybridization signals in the ganglion cell layer (GCL) (F-H). The inset in F shows a representative control experiment using "sense" strand RARαb cRNA. Bar = 50 μm.

The RA receptor subtypes RXRγ and RARαb are expressed in the developing zebrafish retina [57,58]. In mouse, RXRγ participates in the regulation of cone opsin expression [41]. To identify the RA receptor type(s) that mediate the RA-induced effects presented above, the retinal expression patterns of RXRγ and RARαb were determined between 36 and 72 hpf, the period of extensive proliferation and differentiation of photoreceptor cells, and during which exogenous RA influences photoreceptor fate (the present study) and differentiation [31,52]. *In situ *hybridization for RXRγ mRNA revealed strong signals for control embryos at 36 hpf, particularly along the ventronasal side of the choroid fissure (Figure 10A, arrow). Ventrotemporal expression was very low or nonexistent (data not shown). RXRγ also was expressed in cells outside this region including cells along the outer edge of the retina, adjacent to the RPE (Figure 10A, arrowheads), a region containing mitotic progenitors cells (Figure 7; see also [59]). The pattern of RXRγ expression is similar, though more widespread, in embryos examined at 48 and 55 hpf (Figure 10B, C). Note that at 48 hpf, the RXRγ^+ ^outer edge of the retina was becoming laminated and populated with cells that will differentiate as photoreceptors, though it also contained proliferating progenitors ([1]; see also Figure 7A). In embryos examined at 72 hpf, RXRγ expression in the ONL was restricted to the region near the CGZ, and was found in cells at the outer edge of the CGZ (Figure 10D, arrows), suggesting a continuing function for RXRγ in the generation and differentiation of photoreceptors beyond the embryonic period (Figure 10D, arrows). In addition, several cells in the INL expressed RXRγ (Figure 10D, arrowheads). These RXRγ^+ ^cells were present in a distribution similar to the expression pattern of YFP transgene in embryos treated with RA at 72 hpf and examined at 75 hpf (Figure 10F).

Expression of RARαb mRNA was detected at 36 hpf in the ventral retina, adjacent to the lens (Figure 10E, arrows). At later developmental times (48 and 55 hpf), this expression domain expanded throughout the developing GCL (Figure 10F-H; Figure 10F inset: RARαb sense probe). In embryos examined at 72 hpf, RARαb was expressed in the GCL and in a diffuse pattern in the remainder of the retina, including the ONL (Figure 10H).

### Knockdown of RARαb alters RA signaling and reduces the number of rods in the retina

**Figure 11:**
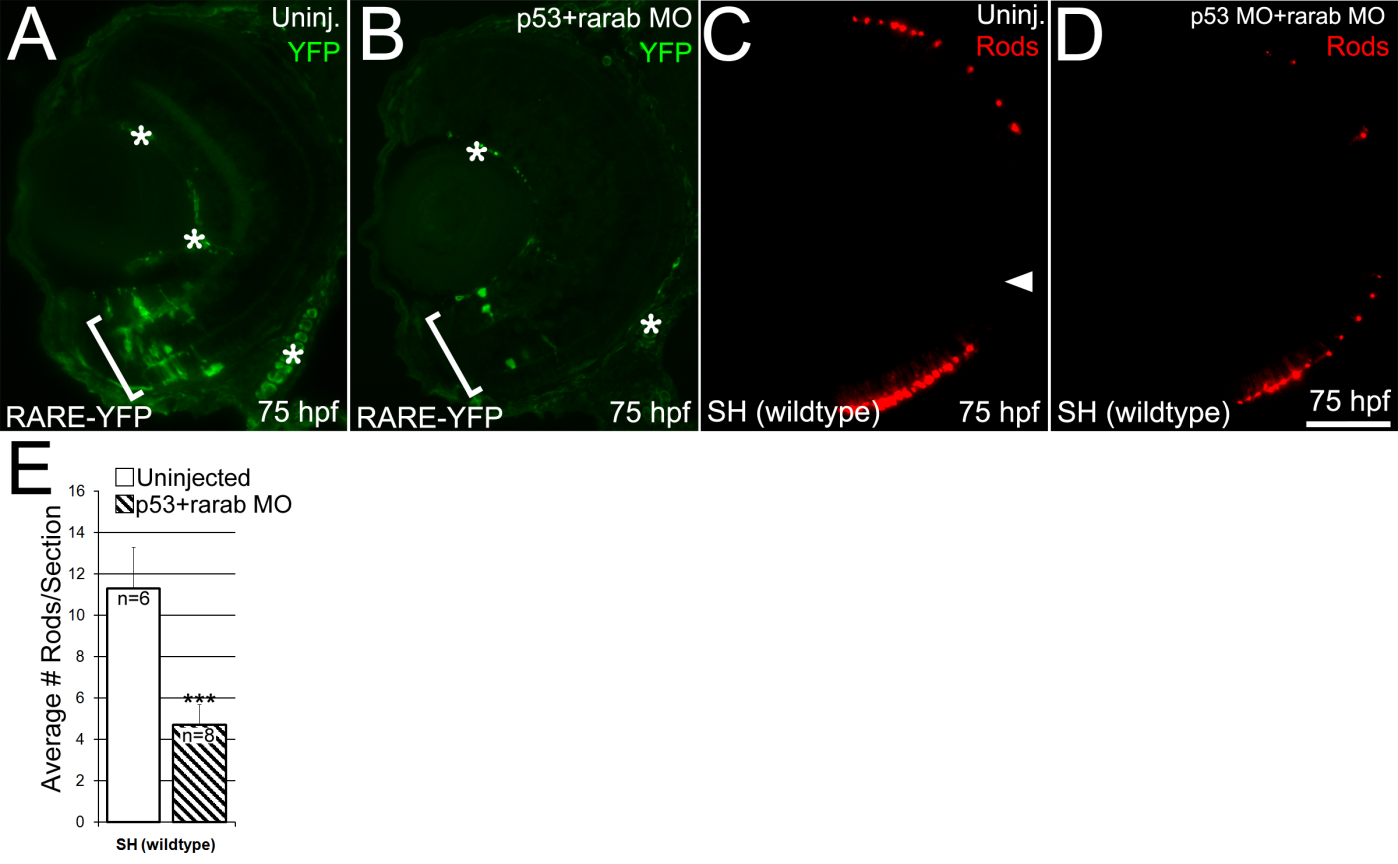
**Endogenous retinoic acid signaling and production of rods is reduced by knockdown of RARαb expression**. (A, B) RARE-YFP embryos at 75 hpf. YFP expression was detected on embryos sectioned at 4 μm using an anti-GFP antibody. (A) In uninjected RARE-YFP embryos, there is robust expression of YFP in cells in the ventral retina (bracket). Non-specific antibody staining is indicated by asterisks. (B) RARE-YFP embryos injected with the *rarαb*/p53 MO show a marked reduction in the number of cells expressing YFP in the ventral retina (bracket). (C, D) Wildtype (SH) embryos at 75 hpf. (C) Tissue section from uninjected wildtype embryo labeled with a rod opsin antibody (1D1). Arrowhead: optic nerve head. (D) Injection of the *rarαb/p53 *MO into wildtype embryos results in reduced expression of 1D1 rod opsin antigen. (E) Numbers of rod photoreceptors were quantified by counting them on sections (see Methods). Wildtype embryos injected with the *rarab/p53* MO (n = 8, average 8 sections per eye) at 75 hpf had a significant reduction in the number of rods per section compared to uninjected embryos (n = 6, average 10 sections per eye); p < 0.5, Student's T-Test). Bar = 50 μm.

The expression patterns for RXRγ and RARαb are consistent with potential functions for these receptors in mediating the effects of RA on photoreceptor identity and differentiation. To gain further insight into the roles of endogenous RA signaling, and to test the hypothesis that RARαb mediates the effects of endogenous RA signaling on photoreceptors, a morpholino oligonucleotide against the RAR subtype RARαb [60] was injected into one-cell stage embryos carrying RARE-YFP. In all of these experiments we co-injected an antisense morpholino against *p53*, in order to suppress MO-related cell death [54,60]. Embryos injected with the *rarαb*/*p53 *MO combination will be referred to as RARαb morphants. RARαb knockdown resulted in a large reduction in the number of cells expressing YFP in the ventral retina at 75 hpf (Figure 11, bracket, compare A to B), suggesting that RARαb is required for some of the endogenous RA signaling within the developing retina. The effects of RARαb knockdown on rod photoreceptor development were then evaluated. Embryos from our wildtype (SH) line were injected with the *rarαb*/*p53 *MO combination and retinal tissue sections examined at 60 hpf with an antibody to rod opsin (1D1). Rods were quantified by determining on a sample of tissue sections the average number of rods in the central and dorsal retina per section (see Methods). In these RARαb morphants knockdown of RARαb caused a significant reduction in the average number of rods per section (Figure 11C, D, E, p < 0.001). These results suggest that knockdown of RARαb by antisense MO results in a partial knockdown of RA signaling in the retina, which in turn affects the generation of rod photoreceptors.

The expression of additional markers for retinal cell differentiation was also tested on RARαb morphant retinas. There were no significant changes in the numbers and distribution of zpr1^+ ^red and green cone cells (Table 6) or islet-1^+ ^cells (data not shown), indicating selective effects on rods and not a general developmental delay. Together these results suggest that development of rod photoreceptors is particularly sensitive to RARαb knockdown.

**Table 6:** Effect of RARαb knockdown on red/green cone differentiation

Treatment group	Average ZPR1 score
ZPR1 examined at 75 hpf:	
uninjected	1 ± 0
*rarαb *MO	1 ± 0
	
ZPR1 examined at 60 hpf:	
*rarαb+p53 *MO, DMSO	0.38 ± 0.44
*rarαb+p53 *MO, RA	0.35 ± 0.24

### RARαb may mediate the effects of exogenous RA on rod development

**Figure 12:**
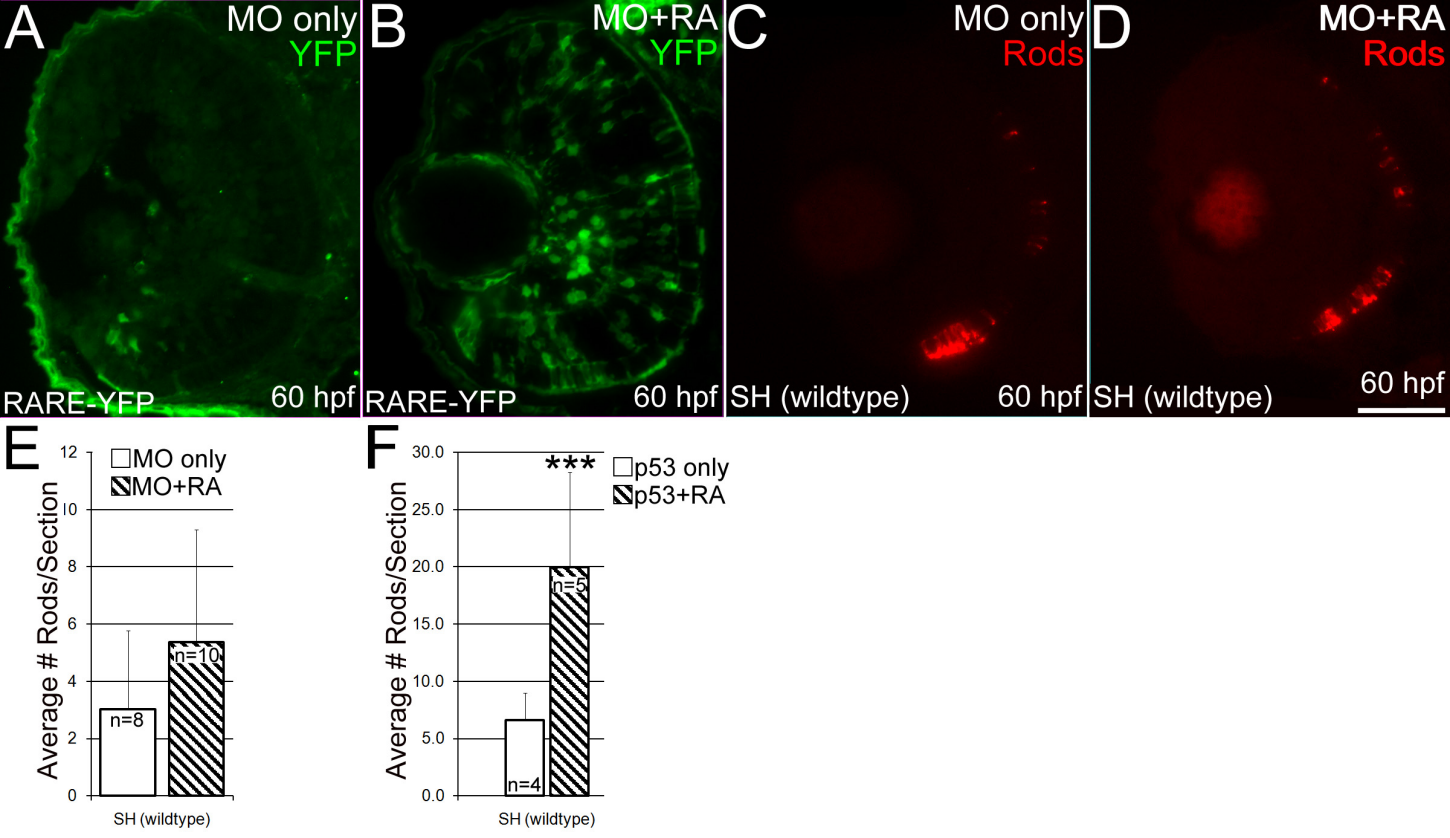
**The effects of exogenous retinoic acid on RARαb morphants**. (A, B) RARE-YPF embryos injected with *rarαb*/*p53 *MO and fixed at 60 hpf. YFP expression was detected on 4 μm cryosections using an anti-GFP antibody. (A) RARE-YFP embryos injected with the *rarαb*/*p53 *MO show reduced expression of YFP in the ventral retina. (B) RARE-YFP embryo injected with *rarαb*/*p53 *MO and treated with RA beginning at 36 hpf. Upregulation of YFP expression is evident in cells across all the retinal layers. (C, D) Wildtype (SH) embryos injected with *rarαb*/*p53 *MO and fixed at 60 hpf, sectioned at 4 μm, and labeled with an antibody to rod opsin (1D1). (C) Embryo injected only with *rarαb*/*p53 *MO. (D) Embryo injected with *rarαb*/*p53 *MO and treated with RA beginning at 36 hpf. (E) Numbers of rod photoreceptors were quantified by counting them on sections (see Methods). Statistically significant differences between DMSO and RA-treated groups were not detected in wildtype RARαb morphants. (DMSO group n = 8, average 9 sections per eye; RA group n = 9, average 8 sections per eye) (n = 13, average 8 sections per eye). Bar = 50 μm. (F) Numbers of rod photoreceptors per section in embryos treated with the *p53 *MO only, and exposed to DMSO (n = 4, average 9 sections per eye) or RA (n = 5, average 8 sections per eye), and examined at 63 hpf. In this experiment RA treatment results in significantly higher numbers of rods. Bar = 50 μm.

We next tested the hypotheses that RARαb is required for 1) increases in retinal RA signaling, and 2) increases in rod development in response specifically to exogenous RA. RARE-YFP embryos were injected with the *rarαb*/*p53 *MO combination at the one-cell stage, were treated with RA beginning at 36 hpf, and examined at 60 hpf for YFP expression and photoreceptor development. RARαb knockdown resulted in a reduction in YFP expression in the retina (Figure 12A, see also Figure 11B). Treatment of RARE-YFP, RARαb morphants with RA at 36 hpf resulted in upregulation of YFP expression in all the layers of the retina by 60 hpf (Figure 12B), suggesting that RARs (or RXRs) in the retina other than RARαb are capable of mediating increased signaling in response to exogenous RA.

Rod photoreceptor development was then assayed in SH (wildtype), RARαb morphants treated with DMSO (Figure 12C') or with RA (Figure 12D'). RA-treated RARαb morphants did not display a statistically significant increase in the average number of rods per section as compared to those treated with DMSO (Figure 12E). The distribution of cone photoreceptors, as assessed by zpr-1 staining, was also not notably different in RA-treated vs. DMSO-treated RARαb morphants (data not shown). Wildtype embryos injected with the *p53 *MO alone and treated with RA at 36 hpf showed a significant increase in the average number of rods per section (Figure 12F), indicating that p53 knockdown did not interfere with a robust effect of exogenous RA on rod development. Collectively these results support a role for RARαb in mediating effects of exogenous RA on rods.

## Discussion

### Sustained high levels of RA signaling modify photoreceptor fate

Treatment of zebrafish embryos with 0.3 μM RA beginning at the time of retinal neurogenesis (36 hpf) resulted in increased densities of rod photoreceptors and correspondingly decreased densities of cone photoreceptors. Local and global pattern attributes (as measured by NND, DRP, and quadrat analyses) of the enhanced rod mosaic and the depleted red cone mosaic collectively suggested that the retinal progenitor population competent to generate photoreceptors, generated more rods, possibly at the expense of red cone photoreceptors. Assessment with other rod and cone-specific markers supported this hypothesis. Alternative hypotheses, such as accelerated/decelerated differentiation of specific cell types, widespread but selective cone cell death, or the generation of photoreceptors of mixed phenotype, were either discounted or poorly supported. A parsimonious explanation for these results is that sustained RA treatment beginning at the time of retinal neurogenesis influences retinal progenitors and photoreceptor precursors, favoring a rod fate over a cone fate, with the spatial positions of the 'missing' cone photoreceptors being anomalously occupied by additional rods.

Several transcription factors have been shown to alter the fates of rods vs. cones. In mouse, absence of either NRL, or NR2E3, results in a retina with photoreceptors expressing only cone markers and having ambiguous photoreceptor morphologies [61-63], and there is evidence that NRL activates NR2E3 to suppress the development of cones [64]. Two other nuclear receptors, RXRγ and TRβ2, are required to suppress expression of the S-cone opsin in mice, favoring the production of cones expressing M-opsin [65,66]. A compelling example of photoreceptor fate manipulation by a single transcription factor is provided by the zebrafish *tbx2b *mutant, in which there is a replacement of UV cones with rod photoreceptors, and the 'new' rods have an entirely unambiguous identity although they occupy the spatial positions of UV cones within the photoreceptor mosaic [13]. *Tbx2b *is expressed in the developing retina well in advance of photoreceptor neurogenesis, suggesting that intrinsic factors controlling photoreceptor fate may exert their effect on progenitor cells rather than upon immediately postmitotic precursors.

The present study now provides evidence that an extracellular signal - RA - can influence the fate of retinal progenitor cells in the zebrafish. Somewhat similar to the situation with *tbx2b*, RA has these effects only when administered in advance of photoreceptor neurogenesis. However, sustained high levels of RA signaling, up to and including the time of photoreceptor terminal mitosis, are required. This finding, together with evidence for induced RA signaling within proliferative cells and new rod photoreceptors, suggests that the developmental trajectory of retinal progenitors can be influenced by extrinsic as well as intrinsic factors. It is interesting that only one other 'extracellular' signaling mechanism has been demonstrated to disrupt the formation of the photoreceptor mosaic in zebrafish: the Notch signal transduction pathway [67]. These effects were obtained by treating zebrafish embryos with pharmacological inhibitors of Notch signaling at 24 hpf or earlier, well in advance of photoreceptor neurogenesis.

An unresolved question is the relationship between embryonic rod and cone progenitors [68]. A recent study from our laboratory [11] found that in zebrafish embryos both rods and cones, as well as rod and cone precursors, express an identical suite of "photoreceptor-specific" transcription factors including *rx1, neuroD, crx*, and *nr2e3*, suggesting that additional factors are required to specify rod vs. cone fate. It is possible that the mechanism controlling photoreceptor fate decisions in zebrafish is stochastic, and depends in part on the relative strength of competing local extracellular (and intracellular) cues to impel a progenitor toward the rod program of development. This model is consistent with further results of the present study, demonstrating that RARαb knockdown causes a reduction in endogenous RA signaling and the number of rod photoreceptors without a significant alteration of other retinal cell types.

The local strength of an RA signal therefore may be a factor influencing rod vs. cone cell fate decisions in the zebrafish retina. The enzymes involved in RA synthesis in the vertebrate retina are expressed in ventral and dorsal domains, suggesting endogenous RA exists in a gradient across the retina [28,31,32,69], with its lowest level in the central retina. The use of the RARE-YFP reporter line corroborates that in zebrafish a strong ventral domain of endogenous RA signaling exists [31], which is also the location of the initial patch of rod and cone photoreceptors [3]. A smaller patch of rods forms in the dorsal retina. In contrast, the central retina (in the center between RA gradients), initially develops few rods. Treatment with exogenous RA during retinal neurogenesis may disrupt the endogenous gradients of RA, increasing the number of retinal progenitors that assume a rod photoreceptor fate and decreasing the population available for cone genesis. In RARE-YFP embryos treated with exogenous RA, some of the YFP^+ ^cells that are mitotic are in the position of potential rod progenitor cells (Figure 7), and the numbers of YFP+ rods may correspond to the additional rods in RA-treated retinas (Figure 8). Similarly a loss of RA signaling in RARαb morphants reduces the number of photoreceptors precursors that ultimately assume a rod fate.

### Pleiotropic effects of prolonged high levels of RA signaling

The RA treatment used in this study resulted in a complex retinal phenotype, with effects on photoreceptor fate as discussed above, but also on laminar position of photoreceptors, and retinal cell survival. The ectopic photoreceptors in RA-treated retinas (Table 3) may have resulted from the fate-influencing activity of RA, such that cells positioned to become inner retinal neurons instead expressed photoreceptor genes. Surprisingly, many of the ectopic photoreceptors could be labeled with cone-specific markers, suggesting that the cone-to-rod fate influencing effect of RA is limited to cells that ultimately reside in the ONL. Alternatively, RA may affect cell movements of photoreceptor precursors, causing some cells to migrate in a basal direction rather than into the ONL. Related to this speculation is that the effects of RA on retinal cell survival were most pronounced within the INL, further indicating specific effects of RA upon this cell population. Exogenous RA may result in cell death in the INL due to the generation of nonviable cells of abnormal phenotype; alternatively, RA may have some other selectively toxic effect on cells of the INL. It is also possible that the apparent effects of RA on photoreceptor fate are indirectly mediated by a tissue disorganizing outcome of RA toxicity. Indeed, we were unable to assess the effects of RA during retinal neurogenesis independently of RA's effects on retinal cell survival. However, in the RARαb morphants, exogenous RA failed to significantly alter the production of rod photoreceptors, providing compelling evidence that the effects of exogenous RA are mediated at least in part by RA signaling via specific RA receptors.

### Cellular RA signaling in response to exogenous RA

Endogenous RA signaling in the vertebrate retina exists in distinct, separate dorsal and ventral domains [31,34,55], suggesting that any effects of endogenous RA signaling on photoreceptor development outside of these domains must be indirect. In contrast, exogenous RA leads to global changes in photoreceptor gene expression in the retina [31,52], or to changes in the production and positioning of specific photoreceptor cell types (the present study) suggesting that the ability of retinal cells to respond to RA is more widespread. Our results with the RARE-YFP line are consistent with global, as well as direct effects of RA on photoreceptor development, as exogenous RA treatment leads to widespread upregulation of the transgene. Multiple cell types are capable of engaging in RA signaling, including mitotic cells, rod and cone photoreceptors, RPE, Müller glia, and inner retinal neurons. Increases in RA signaling occur on a rapid temporal scale that indicates no requirement for upregulation of signaling machinery. A conclusion from these results is that many cells in the retina possess receptors and coactivators capable of generating a cellular response to RA. A more speculative inference is that presumptive photoreceptors that experience prolonged RA signaling during retinal neurogenesis are driven toward a rod rather than a cone fate.

### Retinoic acid receptors and endogenous RA signaling during retinal neurogenesis

The complete family of RAR and RXR genes in zebrafish has been identified [57,58,70]. In the present study we clarified the retinal expression patterns of RARαb and RXRγ. During retinal neurogenesis, RXRγ mRNA appears in a strong ventronasal domain, and then is expressed in cells of the INL (Figure 10). Later in development, RXRγ mRNA is transiently expressed in outer retina. The RXRγ-expressing cells of the outer retina at 48 hpf likely correspond to retinal progenitors fated to become photoreceptors or Müller glia, and the RXRγ-expressing cells of the outer retina observed at 55 hpf correspond to newly-differentiating rod and cone photoreceptors [1,3]. After formation of the embryonic retina, RXRγ expression becomes limited to the edge of the CGZ (Figure 10D), the major source of new retinal neurons during larval and adult growth [9,71] and within the most recently-generated cells of the ONL. RXRγ is therefore a candidate for mediating RA signaling in retinal progenitor cells and cells of the ONL. Our data are consistent with those obtained from mouse and chick models, where RXRγ is expressed in developing cone photoreceptors [23,24,41]. Interestingly, the apparent peak of expression of RXRγ in the outer retina (55 hpf in zebrafish) precedes photoreceptor opsin expression in the majority of photoreceptors [3] (and see ref. 41), making unambiguous colocalization studies in rods vs. cones unfeasible, and suggesting roles for RA signaling over the time of photoreceptor determination as well as differentiation.

RXRs act as homodimers or as heterodimers with other nuclear receptors such as RARs or thyroid hormone receptors (TRs). Similarities in retinal phenotypes of RXRγ null as compared to TRβ2 null mice led to the suggestion that these two nuclear receptors operate together to influence cone photoreceptor gene expression [41,65]. However, the binding partner(s) for RXRγ in the developing retina are not clearly known. In addition, RARα has been shown to mediate RA signaling in the mouse retina [72,73]. The results of the present study found a zebrafish homologue of RARα, RARαb, is expressed early in neurogenesis in the RGC layer, and at low levels throughout the retina at later stages (Figure 10H). Therefore RARαb is also considered a candidate for regulating endogenous RA signaling in the retina.

Targeted knockdown of RARαb resulted in a reduction, though not absence, of the endogenous expression of reporter in the RARE-YFP line (Figures 11 and 12). This is in agreement with knockout studies of RARα in mice, in which the absence of RARα was associated with the elimination of expression of an RA signaling reporter transgene [72,73]. The role of RARα in retinal cell differentiation is more ambiguous. In mice, knockout of RARα has no effect on retinal morphology or retinal cell differentiation. In the present study using zebrafish, knockdown of RARαb resulted in a significant reduction of the number of rod photoreceptors in the central and dorsal retina. These distinct results in mouse and zebrafish may reflect differential subfunctionalization of the RA receptor subtypes in the two model organisms. Alternatively, the experimental endpoints available in zebrafish (number of differentiating rods) may be more sensitive for the detection of the function of RARα. We suggest that, in the zebrafish, RARαb and an unknown binding partner mediate the activation of a rod neurogenesis program in retinal progenitor cells in response to RA. This mechanism was tested further by treating RARαb morphants with exogenous RA to determine if knockdown would block an increase in the number of rods. Exogenous RA resulted in a widespread upregulation of RA signaling in RARαb morphants, but not in a significant increase in the average number of rods. We interpret this to mean that the RARαb receptor is not essential for mediating RA signaling in response to exogenous RA, but does play a role in regulating rod production in the zebrafish retina.

The presence of retinoid receptors in the developing ONL, together with the capacity of differentiating rods, cones, and progenitor cells to respond directly to exogenous RA, indicates a significant role (or roles) for endogenous RA in regulating photoreceptor development. Defining these endogenous roles has been remarkably elusive. In teleost models, reduction of RA synthesis has been accomplished by knocking down expression of β,β-carotene-15,15'-oxygenase (*bcox*) [74], or of the *apc *gene, which also indirectly reduces RA synthesis [75], or of the *vax2 *transcription factor gene, which alters the distribution of RA-synthesizing enzymes [76]. In each of these models the disruption of RA synthesis results in disruption of photoreceptor morphology and of expression of photoreceptor markers [75-77]. Temporally-selective reduction of RA synthesis by treating embryos with the pharmacological inhibitor citral over the time of photoreceptor differentiation resulted in reduced rod opsin expression [52]. In the present study we have uncovered a role for a specific RA receptor, RARαb, in mediating the effects of endogenous, as well as exogenous, RA upon photoreceptor development. However, we note that, in the RARαb morphants, RARαb is chronically depleted over a protracted developmental period, similar to the situation of chronic RA depletion in *bcox *and *apc *morphants [75,77]. Therefore, while these studies collectively provide compelling evidence that endogenous RA signaling, and specifically RARαb, are required for normal photoreceptor development, interpretation of the phenotypes is complicated by the consideration of early roles for RA signaling in eye formation. In the present study we have used a more targeted approach by knocking down the RARαb receptor, which avoids the effects of a global reduction in RA synthesis. However, an indirect role for RARαb in regulation of rod production cannot be ruled out.

### Dynamic roles for RA during vertebrate retinal development

Overwhelming evidence from several animal models supports numerous functions for RA signaling during the development of the vertebrate eye [25,26,78,79]. A collection of *in vitro *and *in vivo *studies specifically demonstrates important activities of RA signaling with respect to photoreceptor development. These include: a) RA promotes the rod fate at the expense of other, non-photoreceptor retinal cell fates [36,40]; b) RA accelerates or decelerates the rate at which differentiating photoreceptors express specific markers [31,52,80]; c) RA promotes photoreceptor survival [37,81], and d) RA recovers photoreceptor differentiation in ethanol-treated embryos [44]. The results reported here now also support a role for RA in promoting the rod photoreceptor fate at the expense of cone fates. We suggest that temporal shifts in the role of RA signaling, and by implication the functional, molecular targets of the RA signaling machinery, underlie these distinct experimental outcomes. Retinal progenitors competent to generate photoreceptors may assume a transient state of plasticity that can be influenced by extrinsic factors such as RA, or intrinsic factors such as *tbx2b *[13], resulting in altered fate of their progeny. Differentiating photoreceptors may also experience a period of sensitivity to extrinsic factors such as RA, which regulate the rate at which they express photoreceptor-specific genes [31,68]. This model predicts that targets of RA signaling will be at least partially distinct in retinal progenitor cells as compared to differentiating rod and cone photoreceptors. This model is consistent with a molecular mechanism recently demonstrated in mouse retina, in which post-translational modifications of nuclear hormone receptors modulates their activity in a dynamic manner [43]. Our ongoing experiments are aimed at identifying cell-type-selective molecular targets in order to further reveal mechanisms through which RA controls photoreceptor development.

## Conclusions

The principal conclusions of this study are: 1) exogenous RA influences the fates of retinal progenitors when delivered over a sustained developmental period beginning prior to photoreceptor terminal mitosis; 2) exogenous RA also influences photoreceptor laminar position, and causes significant retinal cell death; 3) many cell types within the retina, including those undergoing mitosis, can engage in RA signaling, and therefore are capable of responding directly to RA; 4) two RA receptors, RXRγ and RARαb, are expressed within the embryonic retina in a pattern consistent with roles for these receptors in mediating the effects of RA on photoreceptors; and 5) knockdown of one of these receptors, RARαb, diminishes RA signaling in the retina and causes a specific reduction in the number of rod photoreceptors. These conclusions, together with those of prior studies in the zebrafish and other vertebrates, indicate dynamic, pleiotropic roles for RA signaling during eye and photoreceptor development. We propose a model for zebrafish photoreceptor determination and differentiation that includes shifting states of RA-sensitive plasticity within retinal progenitor cells and developing photoreceptors.

## Methods

### Animals and reagent handling

Zebrafish were maintained in monitored aquatic housing units on recirculating system water at 28.5°C. Our wildtype fish were of a strain originally obtained from Scientific Hatcheries (SH; Huntington Beach, CA). Embryos were collected according to [82], with light onset considered to be zero hours postfertilization (hpf) and embryonic age timed accordingly thereafter. Embryos were kept transparent for analysis by incubating them after 11 hpf in system water containing 0.003% phenothiourea (PTU) to inhibit melanin synthesis [82]. All procedures involving animals were approved by the University of Idaho Animal Care and Use Committee.

The transgenic zebrafish line RGnY was used to identify embryo regions undergoing signaling in response to RA (the gift of Elwood Linney, Duke University). This line is transgenic for a DNA construct consisting of three copies of retinoic acid response elements (RAREs) derived from the mouse RAR-beta gene, a zebrafish basal promoter, an enhanced YFP sequence, an SV40 polyadenylation signal, and a small t intron [55]. The zebrafish RAREs are identical in sequence and function to the mouse RAREs [83]. The endogenous expression patterns of YFP in these fish are consistent with known areas undergoing RA signaling [31,55]. We refer to this line as RARE-YFP.

Stock solutions of all-trans RA (Sigma, St. Louis, MO) were prepared in dimethylsulfoxide (DMSO; Sigma) and stored under nitrogen in the dark at -20°C. Prior to treatment, embryos were manually dechorionated, and then stock solution was added to the water to result in a final concentration of 0.3 μM of RA (DMSO was at a final concentration of 0.1%). Embryos were incubated continuously in reagents, with the incubation solution refreshed every 12 hours unless otherwise indicated. The biologically active concentration of RA used was selected based upon the results of [31,52].

### Antisense morpholino injections

Antisense morpholino oligonucleotides (MO) targeting the translation start site to *rarab *(CCA-CAA-CGT-CCA-CGC-TCT-CGT-ACA-T; [60] and p53 (Gene Tools, Inc.) were resuspended in water. Embryos at the one-cell stage were coinjected with 5 ng of *rarab *MO and 2 ng of MO to *p53 *in order to reduce MO-induced cell death [54]. In this study the combination of the two morpholino oligonucleotides will be referred to as *rarαb/p53 MO*, while embryos injected with the combination will be referred to as RARαb morphants.

The number of rods in the retinas of morpholino-injected or RA-treated embryos was estimated by counting on sections the number of cells labeled with 1D1 antibody. Retinas were sectioned in a radial orientation at 4 μm thickness, and only every fourth section was included in the analysis. In general each retina had 8 to 10 sections scored, with both eyes of a specimen being counted separately. Using this method the average number of rods per section was determined. Rods were counted only in the dorsal and central parts of the retina, omitting rods in the dense ventral patch where it was frequently difficult to distinguish individual cells due to their close packing and strong expression of rod opsin.

### In situ hybridization and immunocytochemistry

Dechorionated embryos were fixed with 4% paraformaldehyde (in phosphate-buffered 5% sucrose) for 1 hr at room temperature, and then washed in phosphate-buffered 5% sucrose. Embryos used for whole mount *in situ *hybridization were dehydrated and stored in 100% methanol at -20°C. Embryos processed for immunocytochemistry were washed in increasing concentrations of sucrose, cryoprotected overnight at 4°C in phosphate-buffered 20% sucrose, embedded and frozen in a 2:1 solution of 20% sucrose: OCT medium (Sakura Finetek, Torrance, CA), and sectioned at 3 to 7 μm, as described in [84].

The zebrafish opsin cDNAs, in pBK-CMV phagemid, were the gifts of Thomas Vihtelic (University of Notre Dame). The cDNAs corresponding to RXRγ and RARαb were the gifts of John Postlethwait (University of Oregon). cDNAs corresponding to zebrafish rod transducin (*gnat1) *and zebrafish cone transducin (*gnat2) *were the gifts of Qin Liu (University of Ohio, Akron). Digoxigenin (dig)-labeled cRNA probes were prepared according to the Genius user guide (Roche, Indianapolis, IN). *In situ *hybridizations were done according to Barthel and Raymond [84]. In brief, tissue was rehydrated and treated with (10 μg/ml) proteinase K, dehydrated, and then hybridized overnight at 56^°^C with 1 mg/ml probe in a hybridization solution containing 50% formamide. Hybridization was visualized by using an anti-dig antibody coupled to alkaline phosphatase (Roche), and a color reaction using the substrate 4-nitroblue tetrazolium chloride. Dual in situ hybridizations for rod and red cone opsin were performed as previously described [11].

The mouse monoclonal antibodies zpr1, zpr2, zpr3, and zrf1 (Zebrafish International Research Center) were used at 1:100 dilution. Mouse monoclonal anti-PKC (Santa Cruz) was used at 1:500. Rabbit polyclonal anti-phosphohistone H3 (Millipore) was used at 1:500. Rabbit polyclonal anti-GFP antibody (Torrey Pines Biolabs) was used at 1:1000. Rabbit polyclonal antibodies to rhodopsin (alternatively referred to as rod opsin), and to red and blue opsins (each used at 1:250) were the gifts of Thomas Vihtelic (University of Notre Dame). The 1D1 antibody was the gift of James Fadool (Florida State University) and was used at 1:100. Immunocytochemistry was performed as described [85]. In brief, tissue sections were blocked for 30 min in 20% goat serum, then incubated with primary antibody overnight at 4-C, washed in phosphate-buffered saline containing 0.05% Triton X-100 (Sigma), then incubated with a Cy3- or fluorescein-conjugated secondary antibody (Jackson Immunoresearch) at 1:200 for 1 hr at room temperature, washed again and coverslips mounted with VectaShield (Vector Labs).

Zpr1 labeling of sections from retinas was quantified qualitatively by placing each retina into one of three scoring categories: 1 (retinas have strong label throughout the entire retina), 0.5 (retinas have weak labeling in the dorsal and central retina), and 0 (Zpr1 labeling is limited to a small ventral patch). For each treatment group an average score was determined.

### Acridine orange staining and TUNEL assay

Cell death was examined *in vivo *using acridine orange. Embryos were incubated in system water containing 5 μg/ml acridine orange for 10 minutes, washed twice with unmodified system water, immobilized with 0.00017% tricaine [82], then embedded on slides in 1% low melting point agarose for viewing by epifluorescence. Cell death was further analyzed using the Roche *in situ *cell death detection kit utilizing terminal deoxyuridine nick-end labeling (TUNEL), followed by a peroxidase-based signal amplification step. A series of cryosections (20-25 per specimen, each section separated by 20 μm to avoid double-counting) were labeled by TUNEL assay and positive cells counted and averaged. Statistical significance was determined using Student's T-Test.

### Photography and statistics

Images were captured using a Leica DMR compound microscope with a SPOT camera system (Diagnostic Instruments). Whole retinas and tissue sections were mounted in glycerol and viewed under Nomarski optics. Fluorescently-labeled tissues were viewed using epifluorescence. In some cases images collected under different optical conditions were superimposed using the 'apply image' function in Adobe Photoshop CS software. Antigen colocalization was assessed in dual immunocytochemistry experiments, within the limits of resolution of our objective lens (1.4 μm), by inspection of single epifluorescence and merged images.

Statistical analyses of significance used in this study (Student's T-Test, ANOVA, Fisher Exact Test, and Wilcoxon Two Sample Rank Sum Test) were performed in the R statistical environment (R, 2010).

### Pattern analysis of photoreceptor mosaics

The data for pattern analysis were generated from images of whole mounted embryonic zebrafish eyes hybridized to opsin probes. An additional dataset for pattern analysis was generated from images of whole mounts hybridized to a combination of rod opsin and red cone opsin riboprobes, detected with distinct fluorescent markers. From these images, square-shaped 3,600 μm^2^-sized areas near the central region of the retina, dorsal to the equator, were selected. Identified, labeled cells were assigned unique, coplanar (x, y) coordinate values by using ImageJ software [86]. Theoretical pattern analysis data were generated from samples of images from five control embryos hybridized to red cone opsin, From these native patterns we generated theoretical patterns representing the replacement of red cones by rods, by randomly removing 25% and 50% of the red cone cells from each pattern of red cones An additional theoretical dataset was generated from samples of images from five control embryos hybridized to both rod opsin and red cone opsin. In this dataset, theoretical patterns representing the replacement of red cones by rods were generated by randomly switching identity of 25% and 50% of the red cone cells to a rod identity. Custom software [50,87,88] was used to analyze quantitatively the pattern analysis data by three different methods: nearest neighbor distance (NND) analysis [47]; density recovery profile analysis (DRP [89] and quadrat analysis [49,50]. Conformity ratios (CRs) were calculated as the mean of the NNDs divided by the standard deviations. CRs are reliable indicators of local pattern regularity, with high CRs corresponding to highly regular patterns [47]. DRP analysis determines a 'mean effective radius' as a measure of the average amount of space around a specific cell type that is not occupied by cells of the same cell type [89]. NND analyses and DRP analyses were applied to control, RA-treated, and theoretical datasets in auto-correlative (between cells of same type) and cross-correlative (between cells of different types) modes, as appropriate. Quadrat analysis, a measure of global pattern, results in a statistical measure (Var/Mean)*(N-1), and from this measure the pattern can be categorized, at a particular criterion level, as being significantly more regular or more aggregated than expected for a Poisson distribution [50]. Statistical significance of the pattern analysis data was determined by ANOVA.

## Authors' contributions

CBS designed and performed all the experiments and contributed to the writing of the manuscript. DAC performed the statistical analysis of photoreceptor pattern. DLS designed the experiments and drafted the manuscript. All authors read and approved the final manuscript.

## Supplementary Material

Additional file 1 **Supplemental tables of pattern analysis data**. Five tables of supplementary pattern analysis data. Click here for file

Additional file 2 **Figure S1**. Effects of a short term exposure to retinoic acid on retinoic acid signaling and rod photoreceptors. Embryos carrying the RARE-YFP transgene were treated with either DMSO (A and C) or 0.3 μM RA (B and D) from 36 to 39 hpf. (A and B) Embryos were processed at 49 hpf as 4 μm cryosections for anti-GFP indirect immunofluoresence. (A) Control embryos exhibiting endogenous transgene expression in the ventral retina. (B) Embryos treated with the 'pulse' of RA show strong ventral labeling, as well as very weak labeling elsewhere in the retina. (C and D) Embryos were processed at 60 hpf for whole mount in situ hybridization with a probe directed against rod opsin mRNA. (C) Control embryos show the normal distribution of rods across the retina. (D) Embryos treated with the RA 'pulse' show a similar distribution and density of rod photoreceptors. Bar = 50 μm. Click here for file

Additional file 3 **Figure S2**. Retinoic acid signaling within a subpopulation of red- or green-sensitive cone photoreceptors in response to prolonged retinoic acid treatment. (A to C) Embryos carrying the RARE-YFP transgene were treated with DMSO (A to C) or 0.3 μM RA from 36 to 60 hpf, and processed as 4 μm cryosections for indirect immunofluorescence with an anti-GFP antibody (green) and the antibody zpr1 which labels both red- and green-sensitive cones. (A) In control embryos red and green-sensitive cones are found widely distributed in the retina. (B) In control embryos, endogenous reporter expression is limited to cells of the ventral retina, (bracket) but found in all retinal layers in that region. (C) Merged panel from A and B showing no colocalization with YFP in the dorsal retina (inset). (D) A retina from an embryo treated with RA. (E) In experimental embryos, the RA treatment leads to widespread expression of YFP, including many cells in the ONL. (F) Merged panel from D and E, showing some cones in the dorsal retina expressing YFP (inset, arrowheads) and many that do not express YFP (inset, arrows). Bar = 50 μm. Click here for file
