# Supplementary material for: Plasticity of photoreceptor-generating retinal progenitors revealed by prolonged retinoic acid exposure
Source: BMC Dev Biol. 2011 Aug 30;11:51. doi: 10.1186/1471-213X-11-51 (PMC3189157; doi:10.1186/1471-213X-11-51)
Supplement: Additional file 1 — Supplemental tables of pattern analysis data. Five tables of supplementary pattern analysis data. [file 1471-213X-11-51-S1.PDF]

Table S1. Pattern analysis from single opsin in situ hybridizations.

Nearest Neighbor Distance (mean NND (μm) ± S.D.)

				60 vs. 75 hpf[d]	
60 hpf	Control	RA	RA vs control[d]	Control	RA
Rods	15.9 ± 4.1	9.7 ± 2.2	↓ **(p<0.001)**	↓ **(p =0.02)**	N.A.
Red cones	5.7 ± 0.5	6.0 ± 0.5	(p=0.17)	(p =0.50)	↑ **(p <0.01)**
Blue cones	7.4 ± 0.5	7.8 ± 0.7	(p=0.17)	(p =0.25)	↓ **(p <0.01)**
UV cones	6.9 ± 0.4	7.2 ± 0.4	(p=0.19)	N.A.	N.A

75 hpf	Control	RA	RA vs control[d]
Rods	10.7 ± 1.0	N.D.[a]	N.A.
Red cones	5.8 ± 0.5	7.2 ± 1.0	↑ **(p<0.001)**
Blue cones	6.8 ±1.3	6.9 ± 0.7	(p=0.96)
UV cones	N.D	N.D.	N.A.

Conformity Ratio (mean NND/S.D)

				60 vs. 75 hpf[d]	
60 hpf	Control	RA	RA vs control[d]	Control	RA
Rods	4.0 ± 1.2[b]	3.5 ± 1.4[b]	(p=0.44)	(p =0.1)	N.A.
Red cones	7.4 ± 1.0[c]	5.2 ± 1.4[c]	↓ **(p<0.001)**	↓ **(p< 0.001)**	↓ **(p <0.001)**
Blue cones	7.7 ± 1.4[c]	6.4 ± 1.0[c]	↓ **(p=0.03)**	↓ **(p =0.02)**	↓ **(p <0.01)**
UV cones	7.4 ± 1.2[c]	5.1 ± 1.1[c]	↓ **(p<0.001)**	N.A	N.A

75 hpf	Control	RA	RA vs control[d]
Rods	5.1 ± 0.8[b]	N.D.[a]	N.A.
Red cones	5.8 ± 0.7[c]	3.1 ± 1.0[c]	↓ **(p<0.001)**
Blue cones	6.5 ± 0.7[c]	4.7 ± 1.2[c]	↓ **(p<0.001)**
UV cones	N.D.	N.D.	N.A.

Effective Radius (R_{eff} in μm ± S.D.)

				60 vs. 75 hpf[d]	
60 hpf	Control	RA	RA vs control[d]	Control	RA
Rods	9.3 ± 1.4	7.1 ± 1.4	↓ **(p<0.01)**	(p =0.98)	N.A.
Red cones	4.8 ± 0.5	4.9 ± 0.7	(p=0.75)	(p =0.71)	(p =0.45)
Blue cones	6.3 ± 0.5	6.8 ± 0.8	(p=0.12)	(p =0.23)	↓ **(p <0.01)**
UV cones	5.7 ± 0.6	6.2 ± 0.7	(p=0.13)	N.A	N.A.

75 hpf	Control	RA	RA vs control[d]
Rods	9.3 ± 1.5	N.D.[d]	N.D.[†]
Red cones	4.9 ± 0.6	5.4 ± 2.0	(p=0.38)
Blue cones	5.7 ± 1.2	5.4 ± 1.1	(p=0.47)
UV cones	N.D.	N.D.	N.A.

[a] Rod density was so high as to preclude accurate counting.
[b] Unable to reckon significance of CR due to low number of rods in sample (Cook, 1996).
[c] CR value indicates pattern shows statistically significant regularity (p>0.001; Cook, 1996).
[d] By ANOVA.
N.D. Not done.
N.A. Not applicable.

Table S2. Pattern analysis of theoretical patterns from single opsin in situ hybridization.

Theoretical Red Cone Loss (60 hpf)

	Control	25%	50%	Statistical Comparison[d] 25% vs. Cont.	50% vs Cont.	25% vs. RA	50% vs. RA
NND[a]	5.7 ± 0.5	6.3 ± 0.7	7.1 ± 1.3	(p = 0.13)	↑ (p = 0.06)	(p = 0.39)	(p = 0.12)
CR[b]	7.4 ± 1.0	5.5 ± 1.3	4.1 ± 1.1	↓**(p = 0.03)**	↓**(p < 0.001)**	(p = 0.67)	(p = 0.13)
R_{eff}[c]	4.8 ± 0.5	4.9 ± 0.4	5.3 ± 0.8	(p = 0.75)	(p = 0.20)	(p = 0.96)	(p = 0.29)

[a] Mean Nearest Neighbor Distance (µm) ± S.D.
[b] Conformity Ratio (mean NND/S.D.). CR values indicate pattern shows statistically significant regularity (p>0.001; Cook, 1996).
[c] Effective Radius (µm) ± S.D.
[d] By ANOVA.

Table S3. Nearest Neighbor Distance (NND) analysis based on double in situ hybridization for both rod and red cone opsins.

Autocorrelative (Rods)		Statistical Significance[a]	
	NND	to control	to RA
60 hpf-control	12± 1.4	-	**p=0.048**
60 hpf-RA	9.7 ± 1.8	**p=0.048**	-
25%[b]	7.8 ± 1.1	**p< 0.001**	**p=0.045**
50%[b]	6.7 ± 0.6	**p< 0.001**	**p=0.003**

Autocorrelative (Red cones)		Statistical Significance[a]	
	NND	to control	to RA
60 hpf-control	6.2 ± 0.5	-	p=0.056
60 hpf-RA	7.0 ± 0.8	p=0.056	-
25%[b]	6.8 ± 0.5	**p=0.04**	p=0.64
50%[b]	7.3 ± 0.6	**p=0.005**	p=0.42

Cross Correlative Red cone to Rod		Statistical Significance[a]	
	NND	to control	to RA
60 hpf-control	9.5 ± 1.8	-	**p=0.05**
60 hpf-RA	8.2 ±1.5	**p=0.05**	-
25%[b]	7.4 ±0.8	**p=0.02**	p=0.51
50%[b]	6.5 ± 0.5	**p=0.002**	**p=0.01**

Cross Correlative Rod to Red cone		Statistical Significance[a]	
	NND	to control	to RA
60 hpf-control	5.8 ± 0.44	-	p=0.10
60 hpf-RA	6.3 ±0.35	p=0.10	-
25%[b]	6.4 ± 0.7	p=0.13	p=0.64
50%[b]	7.6 ± 1	**p=0.002**	**p=0.008**

[a]ANOVA
[b]Theoretical patterns based on changing 25% or 50% of red cones in the native control patterns to rod identity.

Table S4. Conformity Ratio (CR) analysis (mean NND/S.D.) based on double in situ hybridization for both rod and red cone opsins.

Autocorrelative (Rods)	CR	Statistical Significance[a] to control	to RA
60 hpf-control	4.2 ± 1.1[c]	-	p=0.55
60 hpf-RA	3.8 ± 1.2[c]	p=0.55	-
25%[b]	3.1 ± 0.4[d]	**p=0.047**	p=0.23
50%[b]	3.8 ± 0.4[d]	p=0.39	p=0.98

Autocorrelative (Red cones)	CR	Statistical Significance[a] to control	to RA
60 hpf-control	5.0 ± 1.3[d]	-	p=0.15
60 hpf-RA	3.9 ± 1.0[d]	p=0.15	-
25%[b]	4.1 ± 0.9[d]	p=0.19	p=0.84
50%[b]	3.0 ± 0.9[c]	**p=0.01**	p=0.14

Cross Correlative Red cone to Rod	CR	Statistical Significance[a] to control	to RA
60 hpf-control	2.7 ± 0.7[d]	-	p=0.78
60 hpf-RA	2.8 ± 0.2[d]	p=0.78	-
25%[b]	3.2 ± 0.6[d]	p=0.19	p=0.12
50%[b]	4.3 ± 0.5[c]	**p=0.001**	**p<0.001**

Cross Correlative Rod to Red cone	CR	Statistical Significance[a] to control	to RA
60 hpf-control	4.7 ± 0.5[c]	-	p=0.19
60 hpf-RA	4.1 ± 1.1[c]	p=0.19	-
25%[b]	4.2 ± 0.9[c]	p=0.20	p=0.86
50%[b]	3.1 ± 0.5[c]	**p<0.001**	p=0.06

[a]ANOVA
[b]Theoretical patterns based on changing 25% or 50% of red cones in the native control patterns to rod identity.
[c]Unable to reckon statistical significance of CR due to low average number of rods in sample (Cook, 1996).
[d] CR value indicates pattern shows statistically significant regularity (p>0.001; Cook, 1996).

Table S5. Density Recovery Profile analysis, returning the mean Effective Radius (Re_{ff}), based on double in situ hybridization for both rod and red cone opsins.

Autocorrelative (Rods)	R_{eff}	Statistical Significance to control	to RA
60 hpf-control	10.6 ± 0.2	-	**p=0.015**
60 hpf-RA	9.0 ± 1.3	**p=0.015**	-
25%	7.0 ± 2.3	**p=0.003**	p=0.09
50%	5.0 ± 0.6	**p<0.001**	**p<0.001**

Autocorrelative (Red cones)	R_{eff}	Statistical Significance to control	to RA
60 hpf-control	4.7 ± 0.7	-	p=0.56
60 hpf-RA	5.2 ± 1.7	p=0.56	-
25%	5.7 ± 1.5	p=0.17	p=0.58
50%	5.4 ± 1.3	p=0.31	p=0.83

Cross Correlative Red cone to Rod	R_{eff}	Statistical Significance to control	to RA
60 hpf-control	5.4 ± 1.2	-	p=0.47
60 hpf-RA	4.9 ± 1.1	p=0.47	-
25%	5.1 ± 1.2	p=0.73	p=0.70
50%	4.9 ± 1.2	p=0.48	p=1.0

Cross Correlative Rod to Red cone	R_{eff}	Statistical Significance to control	to RA
60 hpf-control	4.9 ± 1.3	-	0.45
60 hpf-RA	5.3 ± 0.6	0.45	-
25%	4.9 ± 1.3	0.98	0.44
50%	5.4 ± 1.3	0.52	0.93

[a]ANOVA

[b]Theoretical patterns based on changing 25% or 50% of red cones in the native control patterns to rod identity.
