# Supplementary figures and images for: Plasticity of photoreceptor-generating retinal progenitors revealed by prolonged retinoic acid exposure
Source: BMC Dev Biol. 2011 Aug 30;11:51. doi: 10.1186/1471-213X-11-51 (PMC3189157; doi:10.1186/1471-213X-11-51)

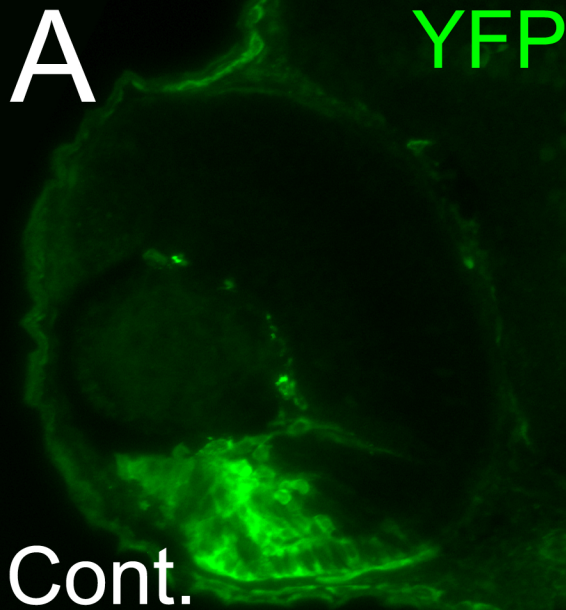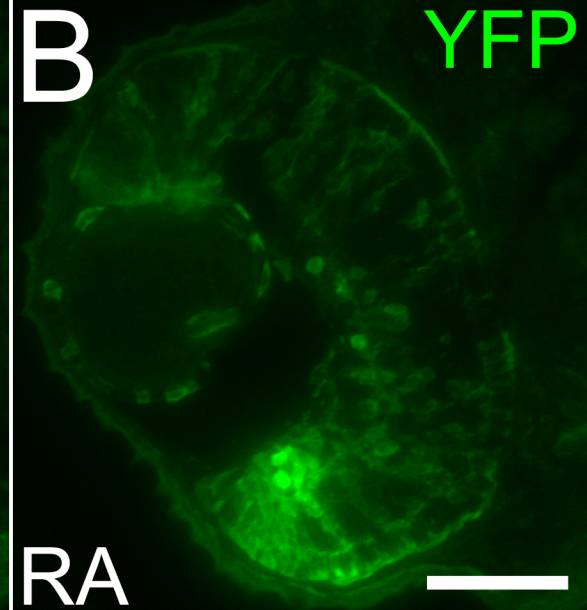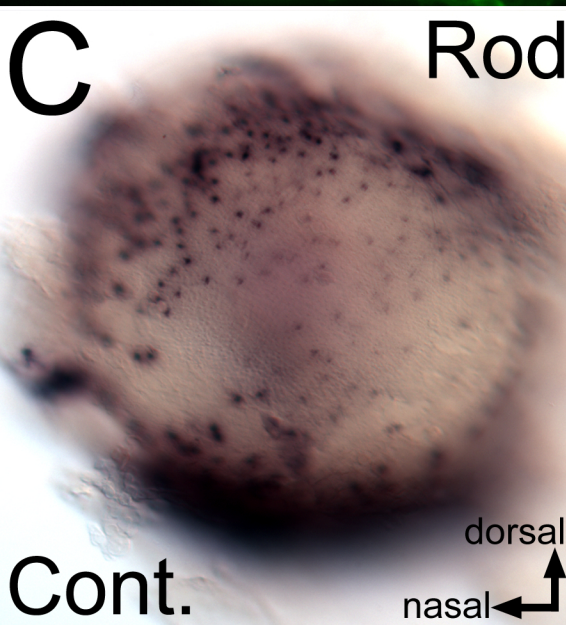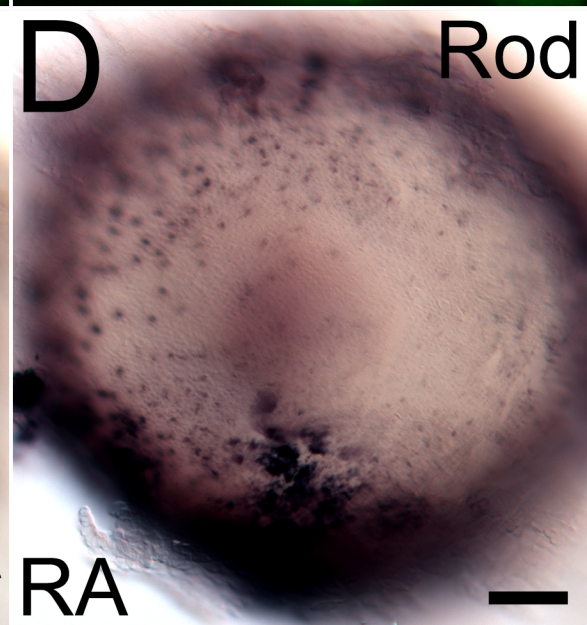

Supplement: Additional file 2 — Figure S1. Effects of a short term exposure to retinoic acid on retinoic acid signaling and rod photoreceptors. Embryos carrying the RARE-YFP transgene were treated with either DMSO (A and C) or 0.3 μM RA (B and D) from 36 to 39 hpf. (A and B) Embryos were processed at 49 hpf as 4 μm cryosections for anti-GFP indirect immunofluoresence. (A) Control embryos exhibiting endogenous transgene expression in the ventral retina. (B) Embryos treated with the 'pulse' of RA show strong ventral labeling, as well as very weak labeling elsewhere in the retina. (C and D) Embryos were processed at 60 hpf for whole mount in situ hybridization with a probe directed against rod opsin mRNA. (C) Control embryos show the normal distribution of rods across the retina. (D) Embryos treated with the RA 'pulse' show a similar distribution and density of rod photoreceptors. Bar = 50 μm. [file 1471-213X-11-51-S2.PDF]

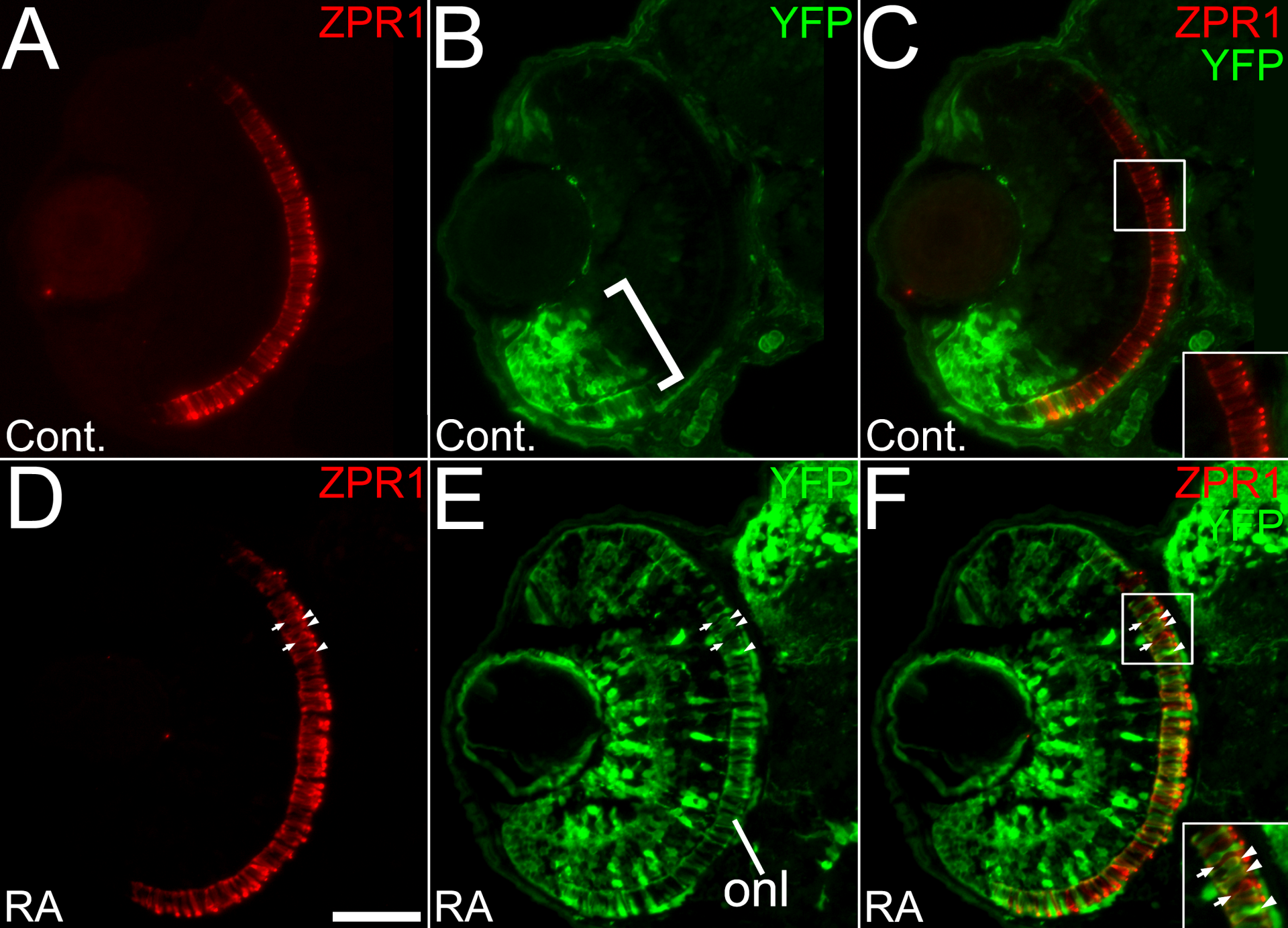

Supplement: Additional file 3 — Figure S2. Retinoic acid signaling within a subpopulation of red- or green-sensitive cone photoreceptors in response to prolonged retinoic acid treatment. (A to C) Embryos carrying the RARE-YFP transgene were treated with DMSO (A to C) or 0.3 μM RA from 36 to 60 hpf, and processed as 4 μm cryosections for indirect immunofluorescence with an anti-GFP antibody (green) and the antibody zpr1 which labels both red- and green-sensitive cones. (A) In control embryos red and green-sensitive cones are found widely distributed in the retina. (B) In control embryos, endogenous reporter expression is limited to cells of the ventral retina, (bracket) but found in all retinal layers in that region. (C) Merged panel from A and B showing no colocalization with YFP in the dorsal retina (inset). (D) A retina from an embryo treated with RA. (E) In experimental embryos, the RA treatment leads to widespread expression of YFP, including many cells in the ONL. (F) Merged panel from D and E, showing some cones in the dorsal retina expressing YFP (inset, arrowheads) and many that do not express YFP (inset, arrows). Bar = 50 μm. [file 1471-213X-11-51-S3.PDF]
